# Determination and validation of design space for mesenchymal stem cell cultivation processes using prediction intervals

**DOI:** 10.1038/s42003-025-08063-2

**Published:** 2025-05-08

**Authors:** Keita Hirono, Yusuke Hayashi, Isuru A. Udugama, Mohamed Rami Gaddem, Kenjiro Tanaka, Yuto Takemoto, Ryuji Kato, Masahiro Kino-oka, Hirokazu Sugiyama

**Affiliations:** 1 Department of Chemical System Engineering, The University of Tokyo, Bunkyo-ku, Tokyo Japan; 2 Department of Basic Medicinal Sciences, Graduate School of Pharmaceutical Sciences, Nagoya University, Tokai National Higher Education and Research System, Nagoya, Aichi Japan; 3 Institute of Nano-Life-Systems, Institutes of Innovation for Future Society, Nagoya University, Tokai National Higher Education and Research System, Nagoya, Aichi Japan; 4 Department of Biotechnology, The University of Osaka, Suita, Osaka Japan

**Keywords:** Computational models, Stem-cell biotechnology

## Abstract

In regenerative medicine, mesenchymal stem cells (MSCs) constitute a promising therapeutic route for many diseases. The current quality-by-design guidelines do not clearly define a framework for MSC production. Here, we suggest and experimentally validate a model-based method to determine design spaces (DSs) for MSC cultivation. A kinetic model used in previous work was employed; part of the experimental data was used to re-estimate the maximum specific growth rate in the kinetic model and then calculate the prediction intervals of this parameter. Subsequently, regions of seeding density and harvesting time where both the upper and lower limits of growth predictions met the acceptable number of cells and confluency with given risk levels were defined as DSs. Finally, the established DS was validated with the remaining data; it allowed better predictions of the cell numbers and confluency under specific cultivation conditions and improved the overall robustness of MSC cultivation processes.

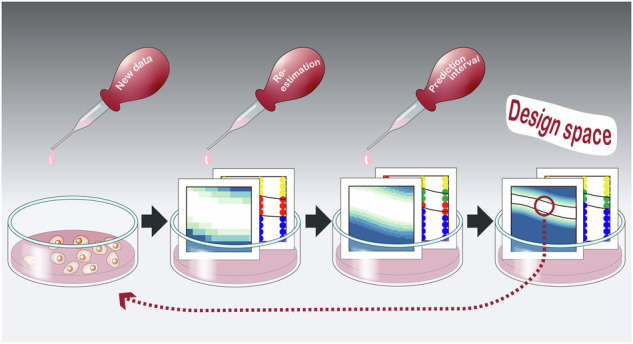

## Abstract (web summary)

This work developed a method to determine and experimentally validate design spaces for mesenchymal stem cell cultivation using mathematical modeling and simulation.

## Introduction

Mesenchymal stem cells (MSCs) are promising for cell therapy applications because of their many therapeutic functions. MSC products have received regulatory approval, and many preclinical/clinical studies on autologous and allogeneic cells^1^ to treat various conditions, such as traumatic spinal cord injury^2^, graft-versus-host disease^3^, and neurological treatments^4^, are ongoing. The demand for high-quality MSCs for potential clinical applications in cell therapy is increasing. For example, there are 2.8 × 10^5^ new cases of Crohn’s disease per year worldwide^5,6^, typically requiring adult single doses of 1.2 × 10^8^ MSCs^7^, suggesting a total demand of >3.0 × 10^13^ MSCs per year for this disease alone. The number of MSCs can be increased through cultivation processes to satisfy cell demands, especially for patient-specific therapy^6^.

The quality of cells in cultivation processes is a critical aspect in terms of phenotypic and functional characteristics of MSCs^6^. In general, for MSCs, cell phenotype is assessed by cell surface biomarker analysis^8^ with the minimum identity criteria^9^, while cell potency is demonstrated by functional assays^10^ regarding immunomodulation^11^, differentiation^12^, and secretion of paracrine factors^13^ as well as self-renewal^14^. Moreover, confluency caused by too many adhesion cells (a high cell density) in a cultivation space, which can decrease cell growth ability^15^, can be a quality attribute of interest. Additionally, cell aging is associated with the accumulation of cell divisions through long-term cultivation, which can decrease the differentiation potential^16^ and be a critical reflector of MSC quality. One major challenge in ensuring the quality of MSCs is accounting for the intrinsic heterogeneity of cells, such as growth dynamics depending on donors or starting cells, toward efficient and reliable cultivation conditions.

Thepharmaceutical industry has addressed the need to satisfy both the anticipated demand growth of MSCs and comply with international regulations^6^. Here, quality by design (QbD)^17^ is a systematic approach to facilitate pharmaceutical development and ensure product quality. Specifically, the concept of a design space (DS)^17^, namely, a multidimensional combination of critical process parameters (CPPs) and critical material attributes (CMAs) to ensure critical quality attributes (CQAs), can be applied to MSC cultivation processes^6^. For example, a DS was shown with two CPPs (the cell seeding density and culture medium change ratio) to satisfy two CQAs (the number of adhesion cells and ammonia concentration)^18^.

For the determination of DSs, traditional experiment-based approaches have been replaced with mathematical model-based methods^19^. Conventional DSs are based on the design of experiments to evaluate the effects of CPPs on CQAs, and their correlation can be statistically analyzed to determine DSs^19^. However, one drawback is the need for a comparatively high number of experiments^19^, which is critical for time-consuming and expensive processes, including MSC cultivation. Thus, supported by experiment-based investigations, model-based methods can enhance the exploration of DSs^19^. Specifically, from the viewpoint of process systems engineering (PSE), modeling is a strong tool for the determination of DSs in pharmaceutical processes, such as those for cultivating cells for cell therapy^20^. In particular, models can be used to determine DSs^19^ with stochastic techniques (e.g., Monte Carlo simulation) that consider system variability (e.g., probabilistic DS on the basis of the minimum acceptable risk^21^). Furthermore, the need for a time-dependent representation of DSs (e.g., dynamic DSs) was discussed on the basis of dynamic models involving ordinary differential equations (ODEs) to address the effects of CPPs on CQAs over time^22^. The state-of-the-art DS determination method for MSC cultivation specifies a dynamic and probabilistic DS, which accounts for both growth kinetics and variability in MSC cultivation^18^. However, the development of methods for DS validation is still in its infancy and is needed for industrial DS determination.

For MSC cultivation processes, a kinetic analysis of growth and metabolism to gain an understanding of the system was established prior to model development^23^. On the basis of these results, a set of ODEs was subsequently developed for cell growth, assuming Monod kinetics considering substrate limitation^24^ and metabolite and contact inhibition^25^. As initial steps of cultivation, cell adhesion and lag time were also integrated with ODEs^18^. Furthermore, spatial growth limitations due to the initial distribution of adhesion cells were addressed by incorporating phase contrast microscopy image data into ODEs^26^. Recently, this model was applied to MSC cultivation processes to find a feasible seeding density and harvesting time to satisfy the given number of adhesion cells and confluency level^26^, which can be used to assess DSs with this model and simulations. However, concerns remain about the reliability of DSs in MSC cultivation processes, as they have yet to be experimentally validated.

In this work, a model-based method is proposed to determine a DS via prediction intervals such that it can be experimentally validated for MSC cultivation processes. Two sets of cultivation experiments were conducted to apply the most recent kinetic model^26^; the first set of experimental data (Exp 1) were used to determine DSs via a published method^18^, whereas the second set of experimental data (Exp 2), which were obtained by three distinct operators, were used for DS validation. However, the calculated DS failed to be validated, with many conditions being incorrectly identified as part of the DS. To overcome this difficulty, we implemented the following four steps: (1) re-estimate the maximum specific growth rate to refine the model using a portion of the Exp 2 data; (2) simulate the upper and lower limits of the growth prediction using 95% prediction intervals of the kinetic model parameter, i.e., the maximum specific growth rate; (3) determine probabilistic DSs with different values of minimum acceptable risk while considering the upper and lower limits of the growth prediction; and (4) validate the obtained DS against the remaining Exp 2 data. The DS was validated with few false positive results, indicating robust conditions for the MSC cultivation process. The presented method can address intrinsic growth variability and thus enhance the applicability of models and the reliability of DSs for the design of industrial MSC cultivation processes.

## Results

### Overall approach for determining and validating the design space

The conventional DS determination method for MSC cultivation processes^18^ was applied with a prior kinetic model^26^ and the data from Exp 1 to determine DSs, as shown in the Supplementary Results (Fig. S1a–c). Here, the model^26^ is a set of ODEs used to simulate previously described CQAs (the number of adhesion cells, $N$, and confluency level, $P$)^26^ as a function of CPPs (the seeding density, ${X}_{{{{\rm{seed}}}}}$, and harvesting time, ${t}_{{{{\rm{h}}}}}$)^26^. Regarding the CQAs, $N$ reflects self-renewal capacity, specifically, the growth rate, which was correlated with cell potency^27^. A high level of $P$ represents cell properties, changing self-renewal capacity^28^ and biomarker expressions^15^. The DS was subsequently defined as sets of CPPs to meet CQAs with a probability equal to or greater than a user-specified minimum acceptable risk^21^, which was set as 90%. The following quality specifications were defined as the same as those in a previous work^26^:

$$\left\{\left(N,P\right)\left|5.0\times {10}^{4}\le N\wedge P \, < \, 0.8\right.\right\}$$

The resulting DS was evaluated with probabilities calculated from the Exp 2 data to ensure the specifications. Additionally, it was possible to classify experimental conditions, i.e., different combinations of ${X}_{{{{\rm{seed}}}}}$ and ${t}_{{{{\rm{h}}}}}$, into four different categories by comparing the probabilistic DS with the Exp 2 data on the basis of metrics developed in prior work^29^, namely, ${CDS}$ (correctly identified feasible condition), $C\overline{{DS}}$ (correctly identified infeasible condition), ${IDS}$ (incorrectly identified feasible condition), and $I\overline{{DS}}$ (incorrectly identified infeasible condition). This validation failed with too many ${IDS}$ conditions incorrectly identified as a part of the DS (Fig. S1d; see Supplementary Results and Fig. S2 for details).

**Fig. 1:**
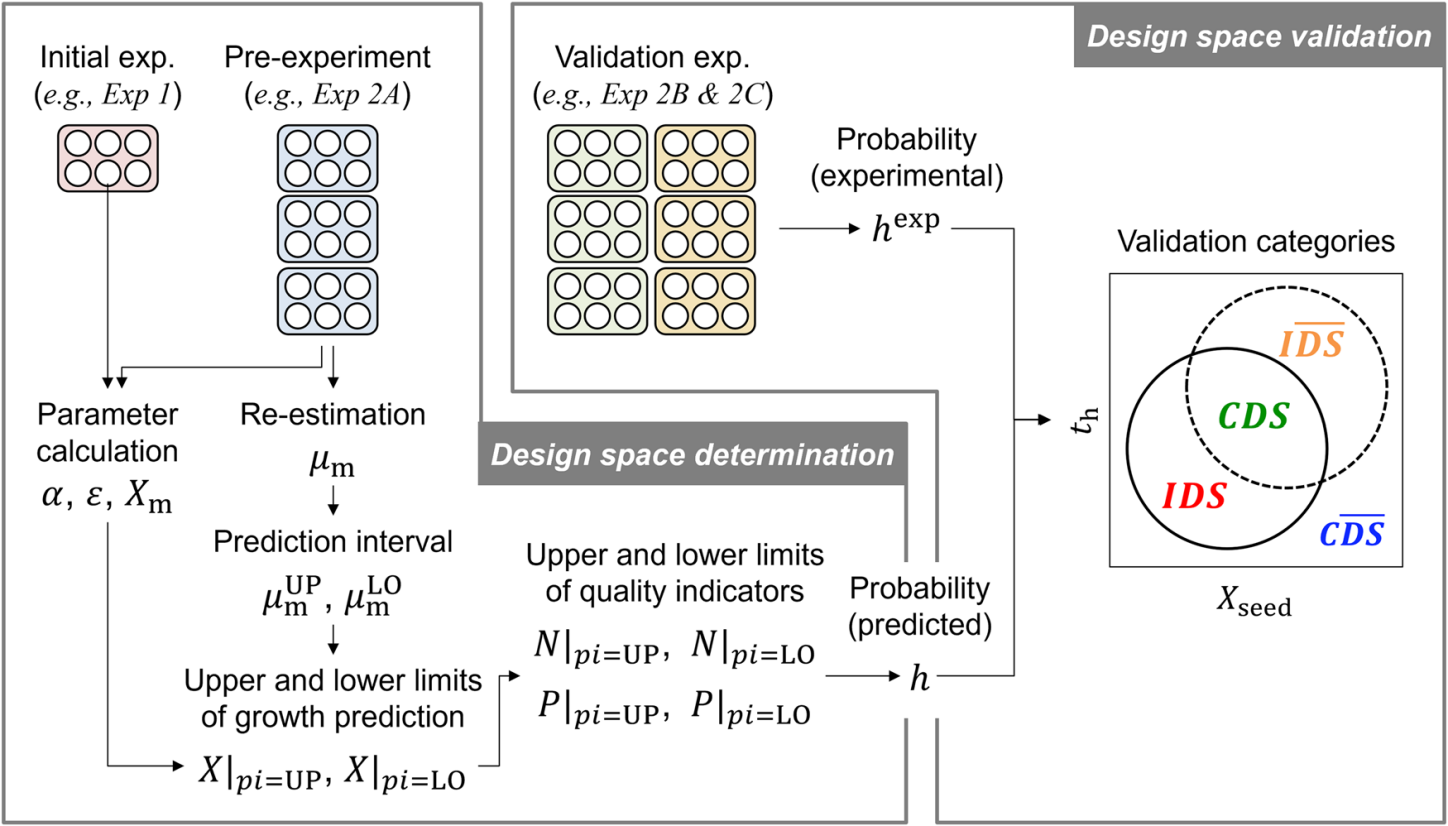
Proposed design space determination and validation workflow. The initial and pre-experimental data were used to determine a design space (DS). The validation experimental data were used to validate the DS with the four categories. The categories that were defined by the DS boundary where the predicted probability was 90% (solid circle) and the experimental results where the experimental probability was 90% (dashed circle). ${{\alpha }}$: adhesion ratio; ${{{\varepsilon }}}$: seeding heterogeneity; ${{{{\mu }}}}_{{{{\rm{m}}}}}$: maximum specific growth rate; ${{{{\mu }}}}_{{{{\rm{m}}}}}^{{{{\rm{UP}}}}}$: upper bound of prediction interval of ${{{{\mu }}}}_{{{{\rm{m}}}}}$; ${{{{\mu }}}}_{{{{\rm{m}}}}}^{{{{\rm{LO}}}}}$: lower bound of prediction interval of ${{{{\mu }}}}_{{{{\rm{m}}}}}$; ${{{{N}}}}_{{{{pi}}}={{{\rm{UP}}}}}$ and ${{{{N}}}}_{{{{pi}}}={{{\rm{LO}}}}}$: upper and lower limits of predicted number of adhesion cells, respectively; ${{{{P}}}}_{{{{pi}}}={{{\rm{UP}}}}}$ and ${{{{P}}}}_{{{{pi}}}={{{\rm{LO}}}}}$: upper and lower limits of predicted confluency, respectively; ${{{{X}}}}_{{{{\rm{m}}}}}$: maximum cell density; ${{{{X}}}}_{{{{\rm{seed}}}}}$: seeding density; ${{{{X}}}}_{{{{pi}}}={{{\rm{UP}}}}}$ and ${{{{X}}}}_{{{{pi}}}={{{\rm{LO}}}}}$: upper and lower limits of growth prediction, respectively; ${{{{t}}}}_{{{{\rm{h}}}}}$: harvesting time; ${{{CDS}}}$: correctly identified feasible condition; ${{{C}}}\overline{{{{DS}}}}$: correctly identified infeasible condition; ${{{IDS}}}$: incorrectly identified feasible condition; and ${{{I}}}\overline{{{{DS}}}}$: incorrectly identified infeasible condition.

To overcome the insufficient validation of the calculated DS, the conventional method^18^ was refined such that the DS could be experimentally validated (Fig. 1). Specifically, some of the Exp 2 data were used to re-estimate ${\mu }_{{{{\rm{m}}}}}$. Moreover, prediction intervals of ${\mu }_{{{{\rm{m}}}}}$ were calculated to generate the lower and upper limits of growth predictions. Finally, the probability of both limits meeting the specifications was calculated to determine DSs, followed by DS validation using the remaining Exp 2 data. The total CPU time for the execution of the whole process in the proposed method was approximately 10 min.

### Re-estimation of the maximum specific growth rate and model validation

**Fig. 2:**
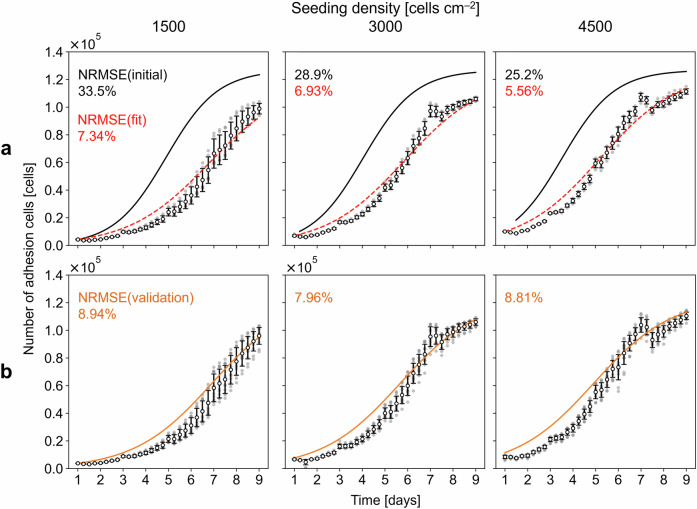
Re-estimation of the maximum specific growth rate results. **a** Model fit to the mean of the experimental number of adhesion cells from Exp 2A for a given seeding density (dashed red lines) compared with the corresponding initial model prediction before re-estimation (black lines). The error bars show the means and standard deviations of the six samples. The normalized root mean square error for the model fit (NRMSE(fit)) and the initial model (NRMSE(initial)) are annotated on the plot. **b** Model validation using the experimental number of adhesion cells from Exps 2B and 2C for a given seeding density (orange lines). The error bars show the means and standard deviations of the 12 samples. The gray scatters represent the individual samples. The normalized root mean square error for the model validation (NRMSE(validation)) is annotated on the plot.

For DS determination, ${\mu }_{{{{\rm{m}}}}}$ was re-estimated with part of the Exp 2 data. Exp 2 was conducted for nine days with seeding densities of 1500, 3000, and 4500 cells  cm^−2^ and was designed to closely mimic Exp 1 but with key differences, including the involvement of three distinct operators (Operators A, B, and C; Exps 2A, 2B, and 2C). Specifically, ${\mu }_{{{{\rm{m}}}}}$ was re-estimated via the data from Exp 2A as a pre-experiment, whereas the data from Exp 2B and 2 C were reserved for validating the model (validation experiments). The fit to the number of adhesion cells measured at different time points for all the individual cultivations in the pre-experiment (i.e., Exp 2A; a total of 18 samples; 3 densities $\times$ 6 replicates) yielded mean and sample standard deviation values of ${\mu }_{{{{\rm{m}}}}}$ of 2.76 × 10^−2^ h^−1^ and 1.46 × 10^−3^ h^−1^, respectively, on the basis of the least squares optimization method (Table S1), with the NRMSE being less than 10%, indicating a better fit to the pre-experimental data than that before the re-estimation (Fig. 2a). Moreover, the NRMSEs between the model predictions obtained using the re-estimated ${\mu }_{{{{\rm{m}}}}}$ and the corresponding measurements in the validation experiment were less than 10% (Fig. 2b), which indicated that the model could be used for DS determination.

### Prediction interval of the maximum specific growth rate and limits of growth prediction

To incorporate the variability of the re-estimated ${\mu }_{{{{\rm{m}}}}}$ into a DS determination, two-sided 95% prediction intervals^30^ of ${\mu }_{{{{\rm{m}}}}}$ were calculated. On the basis of the mean and sample standard deviation of the re-estimated ${\mu }_{{{{\rm{m}}}}}$, both the upper and lower bounds of the prediction interval of ${\mu }_{{{{\rm{m}}}}}$ were set as follows:

$${\mu }_{{{{\rm{m}}}}}^{{{{\rm{UP}}}}}=3.07\times {10}^{-2}\,{{{{\rm{h}}}}}^{-1}$$

$${\mu }_{{{{\rm{m}}}}}^{{{{\rm{LO}}}}}=2.44\times {10}^{-2}\,{{{{\rm{h}}}}}^{-1}$$

**Fig. 3:**
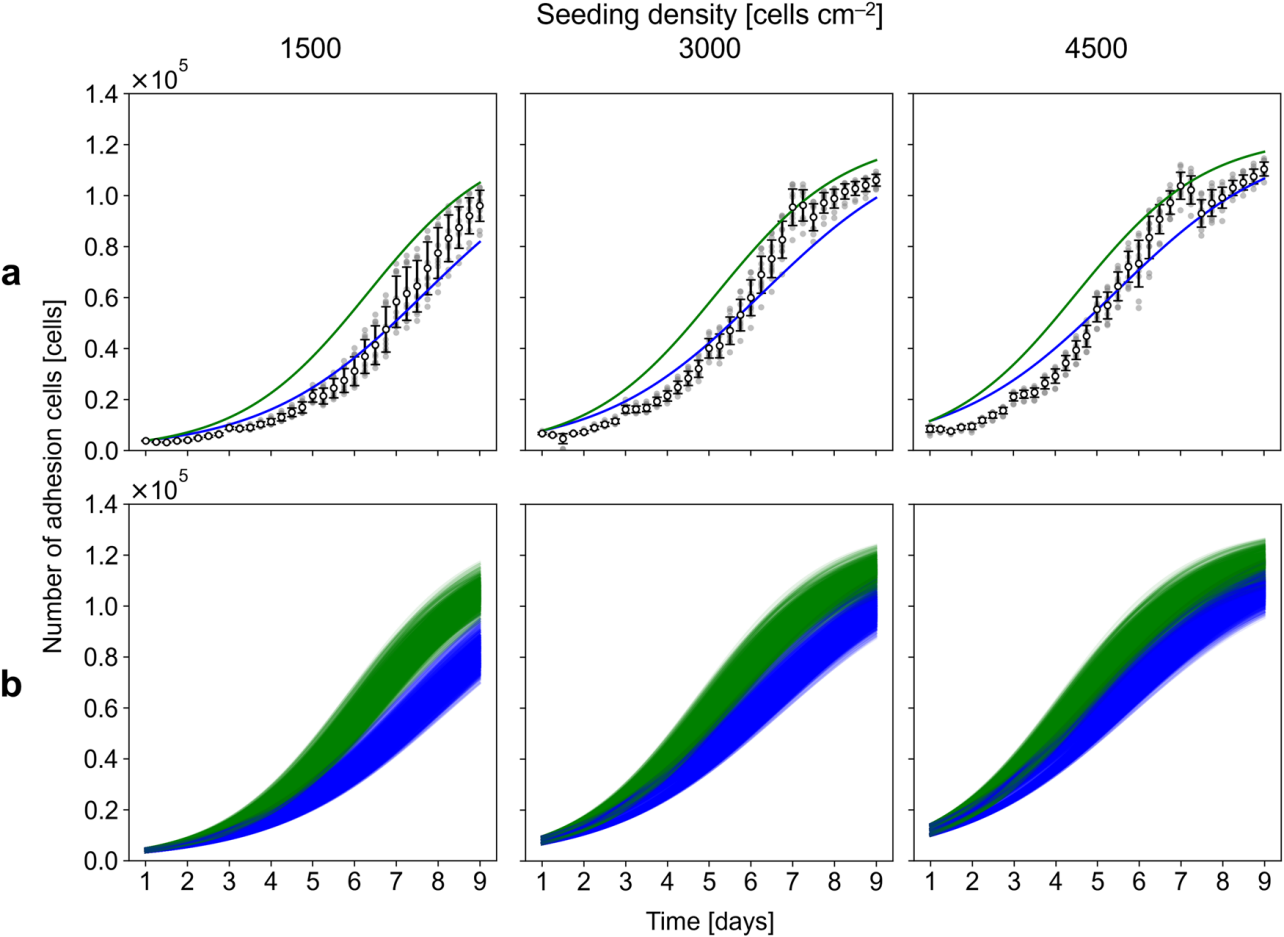
Limits of the growth prediction results. **a** Dynamic simulation of the upper and lower limits with green and blue lines, respectively, for a given seeding density. The error bars show the means and standard deviations of the 12 samples in Exps 2B and 2C. The gray scatters represent the individual samples. **b** Dynamic and stochastic simulations of the upper and lower limits with light green and blue lines, respectively, for a given seeding density.

The calculated ${\mu }_{{{{\rm{m}}}}}^{{{{\rm{UP}}}}}$ and ${\mu }_{{{{\rm{m}}}}}^{{{{\rm{LO}}}}}$ values were substituted into ${\mu }_{{{{\rm{m}}}}}$ (Eq. (6)) to simulate the corresponding upper and lower limits of the model prediction, respectively. Here, the limits of the predicted number of adhesion cells were visualized with the corresponding measurements in the validation experiments (Exps 2B and 2C) (Fig. 3a). These limits indicated the possible growth variation over time to expand upon the average predicted curve (Figs. 2b and 3a). However, the upper and lower limits did not reflect the actual measurements at certain time points. In particular, the lower limits tended to exceed the measurements at earlier time points when there were large errors between the model predictions and the experimental data (Fig. 2b).

In addition to capturing the variability in ${\mu }_{{{{\rm{m}}}}}$, other sources of variation were accounted for by randomly sampling the model parameters on the basis of the Exp 1 and Exp 2 A data. Here, different levels of variation originating from MSC cultivation, as categorized in a prior work^18^, were incorporated, specifically, the seeding heterogeneity, $\varepsilon$ (operation level), the adhesion ratio, $\alpha$ (cell level), and the maximum cell density, ${X}_{{{{\rm{m}}}}}$ (cell level) (Table S2). The resulting variations in the simulated limit were interpreted with underlying biological activities over time. In the cell adhesion phase (around Day 1), low ${X}_{{{{\rm{seed}}}}}$ values resulted in small variations in both the upper and lower limits because ${X}_{{{{\rm{seed}}}}}$ and $\alpha$ mainly affected the number of adhesion cells. In the subsequent growth phase, higher ${X}_{{{{\rm{seed}}}}}$ values yielded smaller variations in both limits because the cell density approached confluency earlier, which decreased the growth rate and then mitigated the variation (Fig. 3b). Here, 1000 iterations were conducted, with good convergence of the simulation results; specifically, the relative standard deviations of the mean of the final number of adhesion cells were less than 0.2% for all ${X}_{{{{\rm{seed}}}}}$ values, which indicated that 1000 iterations would be sufficient.

### Determination and validation of the design space

**Fig. 4:**
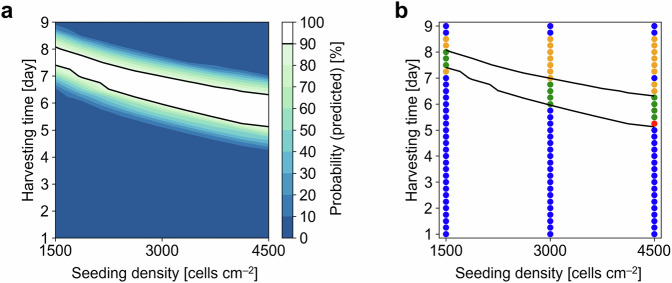
Probabilistic design space and validation results. **a** Design space determination. The contour map shows the predicted probability as a function of seeding density and harvesting time with a design space (DS) boundary where the probability is 90% (black lines). **b** Design space validation. The black lines represent the DS with the resulting categories out of ${{{CDS}}}$, correctly identified feasible condition (green dots); ${{{IDS}}}$, incorrectly identified feasible condition (red); ${{{C}}}\overline{{{{DS}}}}$, correctly identified infeasible condition (blue); and ${{{I}}}\overline{{{{DS}}}}$, incorrectly identified infeasible condition (yellow).

To obtain a DS that can be experimentally validated, the simulated limits based on the prediction intervals of ${\mu }_{{{{\rm{m}}}}}$ were used to calculate the probability of both limits meeting the quality specifications. Following the pre-experimental conditions, ${X}_{{{{\rm{seed}}}}}$ was set from 1500 to 4500 cells cm^−2^ with a numerical increment of 375 cells cm^−2^, whereas ${t}_{{{{\rm{h}}}}}$ was explored during Days 1–9 every 6 h, resulting in 297 combinations of ${X}_{{{{\rm{seed}}}}}$ and ${t}_{{{{\rm{h}}}}}$. The resulting probabilistic DS was visualized as a set of feasible ${X}_{{{{\rm{seed}}}}}$ and ${t}_{{{{\rm{h}}}}}$ with a 2-dimensional contour map. The results suggested that a low ${X}_{{{{\rm{seed}}}}}$ was desirable at the cost of a long ${t}_{{{{\rm{h}}}}}$ (Fig. 4a). For example, at seeding densities of 1500, 3000, and 4500 cells cm^−2^, the cells needed to be cultivated for approximately 7.5–8, 6–7, and 5.25–6.25 days, respectively (Fig. 4a). In contrast, a high ${X}_{{{{\rm{seed}}}}}$ was considered desirable in terms of the number of design choices. Specifically, the feasible range of ${t}_{{{{\rm{h}}}}}$ was narrower at lower ${X}_{{{{\rm{seed}}}}}$ values (e.g., only a 0.5-day range for a seeding density of 1500 cells cm^−2^) than at higher ${X}_{{{{\rm{seed}}}}}$ values (e.g., a 1-day range for seeding densities of both 3000 and 4500  cells cm^–2^) (Fig. 4a). This result was supported by the data shown in Fig. 4a, where the gap between limits was larger in the lower ${X}_{{{{\rm{seed}}}}}$ experiment, indicating higher uncertainty and resulting in a decrease in the calculated probability and consequently a narrower range of feasible ${t}_{{{{\rm{h}}}}}$.

The DS validation was conducted for conditions both inside and outside the DS. Specifically, we evaluated 99 experimental conditions with sets of ${X}_{{{{\rm{seed}}}}}$ values of 1500, 3000, and 4500 cells cm^−2^ and ${t}_{{{{\rm{h}}}}}$ values over Days 1–9 every 6 h (a total of 33 measurement points). For each ${X}_{{{{\rm{seed}}}}}$ and ${t}_{{{{\rm{h}}}}}$, a total of 12 samples were investigated by two operators (Exps 2B and 2C; each analyzing 6 samples) to calculate the probability of the experimental data satisfying the specifications. The probability was subsequently used to categorize the 99 conditions into the four categories defined in Fig. 1. The resulting categories indicated that twelve conditions were included in the DS (those classified as ${CDS}$ and ${IDS}$), among which 11 conditions were confirmed as feasible in the validation experiment (${CDS}$) (Fig. 4b). This result yielded an ${R}_{1}$ (the ratio of conditions included in the DS that were feasible to the total number of conditions in the DS) of 0.917 (Eq. (21)). The conditions classified as ${IDS}$ resulted in many adhesion cells in the simulation; specifically, the lower limit of predictions led to the overestimation of the number of adhesion cells that could still meet the quality specification. The resulting high ${R}_{1}$ value suggested that the DS could serve as a reliable condition for MSC cultivation.

Additionally, other ratios were investigated to evaluate the validation results. From an experimental perspective, 29 conditions were identified as feasible (${CDS}$ and $I\overline{{DS}}$), among which 11 conditions were included in the DS (${CDS}$) (Fig. 4b). From these categories, ${R}_{2}$ (the ratio of the feasible conditions correctly included in the DS to the total number of feasible conditions) was 0.379 (Eq. (22)), potentially because of the conservative prediction of the confluency level. Specifically, the upper limit of predictions indicated that the confluency level did not meet the specification, but the measured confluency did. In contrast, 70 conditions were identified as infeasible in the experiment ($C\overline{{DS}}$ and $I\overline{{DS}}$), among which 69 were correctly identified as being outside the DS ($C\overline{{DS}}$) (Fig. 4b). From these categories, ${R}_{3}$ (the ratio of the number of infeasible conditions correctly discovered by the DS to the total number of infeasible conditions) was 0.986 (Eq. (23)). These resulting low ${R}_{2}$ and high ${R}_{3}$ values suggested that more than half of the conditions required to meet the specifications were not identified, but most of the infeasible conditions were correctly excluded from the DS, which would be beneficial to potential producers, users, and consumers (e.g., patients receiving MSC therapy).

### Effects of prediction intervals on the determination and validation of design spaces

**Fig. 5:**
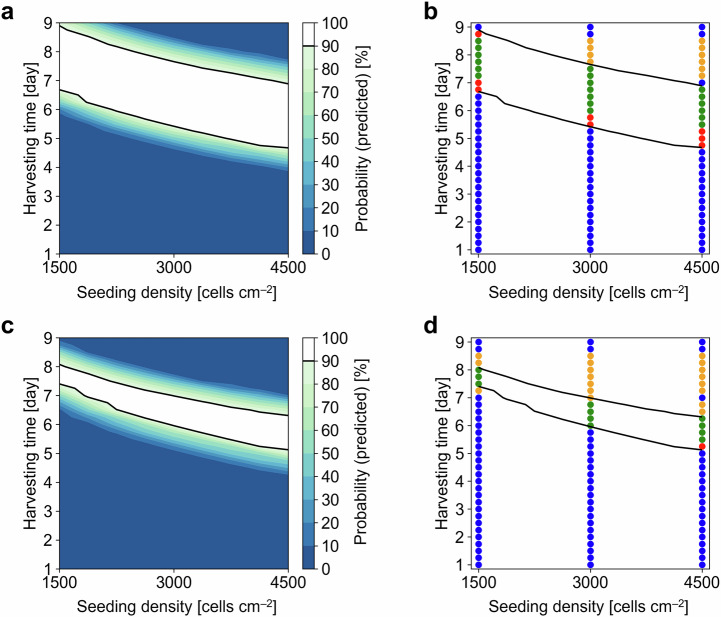
Design space determination and validation results without/with prediction interval calculation. **a** Design space without prediction interval calculation. **b** Validation of the design space without the prediction interval. **c** Design space with prediction interval calculation. **d** Validation of the design space with the prediction interval. Here, the contour map shows the predicted probability as a function of the seeding density and harvesting time. The black lines show the design space with the resulting categories out of ${{{CDS}}}$, correctly identified feasible condition (green dots); ${{{IDS}}}$, incorrectly identified feasible condition (red); ${{{C}}}{{\overline{DS}}}$, correctly identified infeasible condition (blue); and ${{{I}}} {{\overline{DS}}}$, incorrectly identified infeasible condition (yellow).

To investigate the effects of the prediction interval of ${\mu }_{{{{\rm{m}}}}}$ on the determination and validation of the new DS, a reference DS was determined following the re-estimation of ${\mu }_{{{{\rm{m}}}}}$ but without calculating the prediction interval of ${\mu }_{{{{\rm{m}}}}}$. The reference DS included more conditions than those generated with consideration of limits of growth predictions (Fig. 5a, c). However, the reference DS resulted in a lower ${R}_{1}$ than the new DS (Table S3); specifically, the new DS achieved a reduction in the number of ${IDS}$ conditions (Fig. 5b, d). Simultaneously, this reduction yielded a higher ${R}_{3}$ but a lower ${R}_{2}$ because of the tradeoff between the ${IDS}$ and $I\overline{{DS}}$ conditions; namely, the $I\overline{{DS}}$ conditions increased (Table S3). This result confirmed that few conditions were incorrectly identified as part of the DS, suggesting that the prediction interval of ${\mu }_{{{{\rm{m}}}}}$ enabled the DS to represent a conservative estimate of the reliable conditions for MSC cultivation. This reduction in the number of ${IDS}$ conditions would be beneficial for MSC manufacturers because the cultivation conditions can be safely selected within the DS, ensuring that product specifications are met.

### Determination and validation of the design space with different pre- and validation experiments

**Fig. 6:**
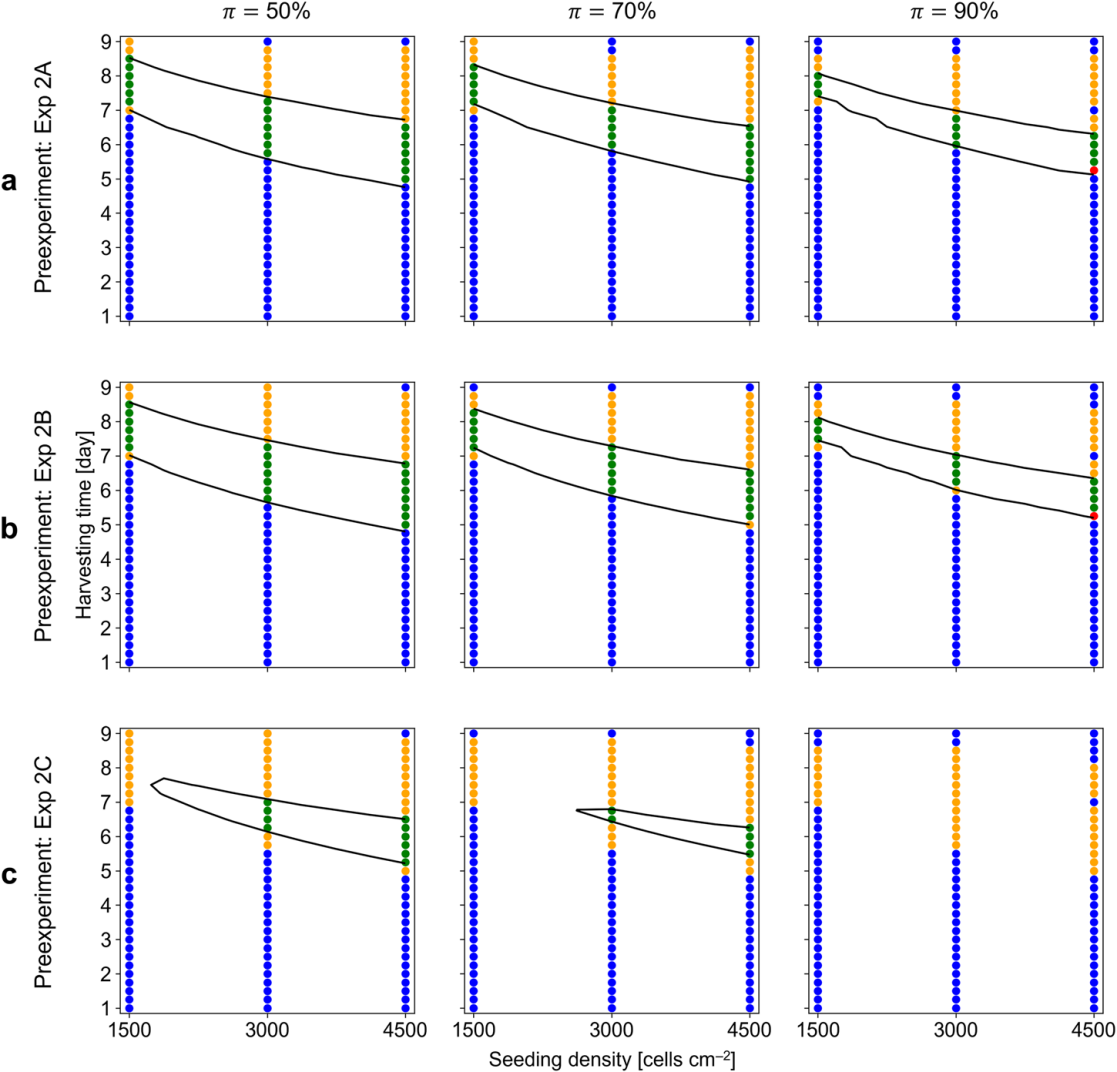
Design space validation results with different pre-experiments. The design spaces were determined via preliminary results and then validated with validation experimental results for a given minimum acceptable risk, ${{{\pi }}}$. The black lines represent the DS with the resulting categories out of ${{{CDS}}}$, correctly identified feasible condition (green dots); ${{{IDS}}}$, incorrectly identified feasible condition (red); ${{{C}}}\overline{{{{DS}}}}$, correctly identified infeasible condition (blue); and ${{{I}}}\overline{{{{DS}}}}$, incorrectly identified infeasible condition (yellow). **a** Exp 2A and Exps 2B and 2C were used for the pre-experiment and validation experiments, respectively. **b** Exp 2B and Exps 2C and 2A were used for the pre-experiment and validation experiments, respectively. **c** Exp 2C and Exps 2A and 2B were used for the pre-experiment and validation experiments, respectively.

To further investigate the applicability and reliability of the new DS, the determination and validation of DSs were extended to different sets of pre- and validation experiments subject to different minimum acceptable risks, $\pi$. Specifically, one experiment from Exp 2 (e.g., Exp 2B) was used for the pre-experiment, whereas the remaining experimental data were used for the validation experiments (e.g., Exps 2C and 2A). Following the re-estimation of ${\mu }_{{{{\rm{m}}}}}$ via pre-experimental data, model validation, and prediction interval calculation (Fig. S3), a DS was determined and then validated for $\pi$ values of 50, 70, and 90%. The resulting DSs indicated that the area covered by the DS narrowed as $\pi$ increased (Fig. S4). This trend was observed for all the pre-experimental data (Fig. S4a–c). Some combinations of $\pi$ and pre-experiments, such as those shown in Fig. S4c, provided no DS (i.e., no white area) because all the calculated probabilities were lower than $\pi$. This occurred because there was a large sample standard deviation for ${\mu }_{{{{\rm{m}}}}}$ in the pre-experimental data (i.e., Exp 2C) that expanded the prediction interval of ${\mu }_{{{{\rm{m}}}}}$ and, correspondingly, the range of the limits of growth prediction. Regardless of the chosen $\pi$ and pre-experimental conditions, these DSs included only a small number of ${IDS}$ conditions (Fig. 6), resulting in high ${R}_{1}$ and ${R}_{3}$ values at the cost of a lower ${R}_{2}$ (Table S4). This result suggested that pre-experiments with little variation in the data, such as Exp 2A and 2B, are preferred if the required $\pi$ is relatively high; this finding is relevant for industrial MSC cultivation process design. Together, these results suggest that the DS could be experimentally validated, ultimately providing reliable conditions for the design of the MSC cultivation process.

## Discussion

A model-based method to determine a probabilistic DS for MSC cultivation processes was presented and validated with experimental data. The originality of this work lies in the creation of a model-based method that combines experimental and numerical procedures to guide the determination and validation of DSs (Fig. 1). The method can provide more clarity to the design of cell cultivation processes, overcoming the inconsistency in model performance due to experimental variabilities such as differences in cell source, operator, and other factors. Such reproducibility issues commonly encountered in cell cultivation^31–33^ could be overcome by applying this method. In this method, the value of the maximum growth rate parameter ${\mu }_{{{{\rm{m}}}}}$ is crucial; hence, there were additional steps for re-estimating ${\mu }_{{{{\rm{m}}}}}$ on the basis of a pre-experiment to increase the model accuracy and, ultimately, its applicability to different MSC cultivation conditions. The re-estimation of ${\mu }_{{{{\rm{m}}}}}$ addressed the discrepancies and variabilities that arose from experiments. For example, the MSCs cultivated in Exps 1 and 2A–2C were seeded from the same lot, but the apparent growth rates measured in Exps 2A–2C were much slower than those in Exp 1. To incorporate such growth variability within a given lot, one set of experimental data of interest (i.e., Exp 2A) was used to re-estimate ${\mu }_{{{{\rm{m}}}}}$, which allowed model validation with the data from Exp 2B and 2C to be successful. This procedure was also used with different permutations of data in the pre-experiment (e.g., Exp 2C) and validation experiments (e.g., Exps 2A and 2B), thus accounting for operator variability; specifically, the relevant parameter re-estimation and model validation resulted in sufficiently small NRMSEs (Fig. S3). These results suggest that the pre-experiment and the re-estimation of ${\mu }_{{{{\rm{m}}}}}$ made the model for DSs more generalizable across cell sources within a single lot. Moreover, the results also addressed inconsistency in cultivation results due to operator variability, which can be of industrial relevance toward commercial MSC manufacturing.

Furthermore, the implementation of a prediction interval for ${\mu }_{{{{\rm{m}}}}}$ supported the discernment of a feasible operation region in the DS while respecting a range of minimum acceptable risk ($\pi$ of 50–90%). The use of the prediction interval of ${\mu }_{{{{\rm{m}}}}}$ is beneficial for incorporating growth variations among cell populations from the same source into process design. For example, upper and lower limits of growth predictions can be generated to incorporate the likely variation on the basis of pre-experimental data into the DS, such that only reliable cultivation conditions are included. This approach could be extended to other pharmaceutical cell cultivation processes that involve growth variations among cell populations from the same source. With increased volume of experimental data, the prediction intervals of ${\mu }_{{{{\rm{m}}}}}$ would converge, which could mitigate statistical uncertainty in simulations to establish more reliable DSs for cultivation processes.

The impact of the sample size on the predictive capacities of the model was evaluated (see Supplementary Results for details). The results of model validation were more susceptible to the sample size than those of model fitting, indicating that a minimum acceptable size was twelve for a given accuracy criterion of 10% (Fig. S5). Furthermore, the impact of these sizes on the DS validation results (Fig. S6) indicated that a total of 18 samples were necessary to yield an ${R}_{1}$ value (i.e., precision) of 90% (Tables S4 and S5). These results suggest that pre-experiments should be designed aiming at the validation of DSs rather than the model itself, which would not only ensure the accuracy of the model but also enhance the reliability of the DSs.

The present model would be useful for various scenes in research, development, and manufacturing of MSCs. In research, for example, the model can be used for uncertainty and sensitivity analysis of experimental parameters (e.g., seeding heterogeneity) of cell cultivation. For manufacturing, the model-based DS could serve as a basis for quantitative decision-making to fulfill a specified product requirement (e.g., dose) subject to timeline constraints (e.g., scheduling).

Given the simplicity of the model and the experiments with a single lot of MSCs, the results presented are concerned with specific cell sources and experimental setups. Cell phenotype can deviate along cultivation in many ways including confluency^15^. To mitigate such deviations, the daily replacement of half of the medium was conducted in the experiment, and thus, it was assumed in the model that the nutrients (e.g., glucose) and metabolites (e.g., lactate) exhibited consistent effects on growth kinetics. Moreover, MSC cultivation is characterized by cell division, contact inhibition^15,25^, initial cell spatial distribution^26^, cell migration^34,35^, morphology^36^, and mechanotransduction^37^. Among these specificities, the model focuses on cell division (i.e., number-based description) with the aim of considering the effects of the initial spatial distribution on the growth. Our prior model^26^ enabled the incorporation of the spatial growth limitation due to seeding heterogeneity (see Fig. 5 in ref. ^26^), which was specific to MSC cultivation, and this was inherited by the present model (see also Fig. 2). The model could serve as a basis for capturing further specificities of MSC cultivation.

The developed procedure (Fig. 1) can be applied to various design cases of MSC cultivation by necessary adjustment with different levels. The common concept here is that the initial experiment (e.g., Exp 1) establishes a baseline of a model for a given lot and experimental setup, while pre-experiments (e.g., Exp 2) aim to quantify and incorporate variability within the lot and setup into the model prediction. The following three scenarios could provide practical guidance for the use of this procedure.

- For MSCs from different batches within the same lot and cultivation condition as this work, only pre-experiments are performed to incorporate growth variability within the lot.
- For MSCs from different lots (e.g., tissue^38^, donor^25,39^) under the same condition as this work, an initial experiment is first conducted for the given lot to establish a baseline for the model under controlled culture conditions. If the model fails to demonstrate the growth kinetics in the initial experiment, model adjustments are needed (e.g., introduction of new parameters). Subsequently, pre-experiments are performed to incorporate growth variability within the lot, while investigating broader conditions (e.g., seeding density, harvesting time), including those relevant to the manufacturing settings.
- Regardless of the source of MSCs, if either the medium composition or the cultivation scale/configuration differs from that employed in this work, a new set of models should be developed. The set of ODEs can be renewed to predict mechanistic cell behavior more rigorously, by adopting, among others, time-dependent metabolism subject to a medium change^18^ and growth kinetics on microcarriers using a bioreactor^40^. An initial experiment is then conducted for the given lot and experimental setup (i.e., medium composition, scale/configuration) to establish a baseline for the model, followed by pre-experiments incorporating growth variability within the lot under the setup.

As a potential limitation of this work, only quality indicators that can be modeled were employed as the CQAs in this method. Consequently, non-modeled CQAs that correlate with the potency of MSCs should be addressed with further analyses or assays toward clinical applications. For example, cell aging^16^ has not been included in the quality specifications used to determine a DS. Cell aging is negligible in the absence of subcultures, as the accumulated number of cell divisions in a single cultivation experiment was smaller than that observed when the aging of bone marrow-derived MSCs occurred^16^. In future work, more experimental results could be explored to enhance the understanding of additional quality indicators and accelerate further computational investigations, improving the robustness of the design of MSC cultivation processes.

## Methods

### Cell culture

Bone marrow-derived MSCs (Lonza Japan, Ltd., Tokyo, Japan; lot number 19TL281098) were thawed, rinsed with MSCGM (Lonza Japan, Ltd., Tokyo, Japan; lot number 0001099016), counted, and then directly seeded in a 6-well plate (353046; Corning Incorporated, Corning, NY, USA). To match the image-analyzed number of cells with the total number of seeded cells, a restricted culture area was created in each well of the 6-well plates (353046, FALCON, NY, USA) using polydimethylsiloxane (PDMS) as described previously^26^. The restricted culture area (17 × 17 mm) was designed to be slightly larger than the total tiling image size (15.3 × 15.3 mm). Exp 1 was designed to collect initial data under controlled conditions to establish a baseline for the kinetic model. MSCs were seeded at a density of 2.0 × 10^3 ^cells cm^–2^ ($n=$ 6) in the restricted area. All operations were conducted by a single operator. Exp 2 aimed to validate the model under broader and more variable conditions by including three operators (Exp 2A, 2B, 2C) using a different batch of MSCs from the same lot as Exp 1. MSCs were seeded at densities of 1.5 × 10^3^, 3.0 × 10^3^, and 4.5 × 10^3^ cells cm^–2^ ($n=$ 6 for each condition) to explore a wider range of seeding conditions. After 1 mL of cells was seeded, the plate was incubated for 1 h to allow cell adhesion. Then, an additional 1 mL of medium was added to bring the total culture volume to 2 mL, ensuring that the liquid surface height was greater than the PDMS height to eliminate the meniscus effect. In Exp 1, all operations were performed by a single operator, and the culture period was 8 days, with 50% of the culture medium replaced every 48 h. In Exp 2, operations were carried out by three different operators (Exp 2A, 2B, and 2C). The cells were cultured for 9 days, and 50% of the culture medium was replaced every 24 h. The culture period was designed to cover not only the early sparse status of the cells but also the confluence status to cover the total culture period to fit the simulation growth curve.

### Image acquisition and processing

Phase contrast microscopy images were automatically acquired every 6 h via BioStation CT (Nikon Corporation, Tokyo, Japan) at 4× magnification (8 × 8 tiling per well, 15.3 × 15.3 mm, 1000 pixels/image). Imaging started 1 h after seeding and continued for 8 to 9 days. Since the tiling image was designed to cover the total cell culture area, we used image processing to count the growth of total seeded cells. Image processing for segmentation and cell counting per image was carried out with the processing pipeline described previously^26^. Image processing and data processing were performed via original codes via Python 3.9.13 with the packages NumPy 1.20.0 and OpenCV 4.4.0.

### Parameter value calculation from experimental data

For each cultivation sample, the adhesion ratio, seeding heterogeneity, and maximum cell density were calculated via the number of adhesion cells counted in the images. Here, the adhesion ratio, $\alpha$, was defined as the ratio of the sum of the number of adhesion cells counted from all the images on Day 1 to the number of seeded cells; the seeding heterogeneity, $\varepsilon$, was the population standard deviation of the normalized number of adhesion cells counted from each image on Day 1^26^; and the maximum cell density, ${X}_{{{{\rm{m}}}}}$, was calculated on the basis of the maximum number of adhesion cells counted from each image from Day 1 to the end of cultivation as follows:

1 $$\alpha =\frac{{\sum}_{i}^{64}{N}_{i}^{{{{\rm{img}}}}}}{S{X}_{{{{\rm{seed}}}}}}$$

2 $$\varepsilon =\sqrt{\frac{1}{64} {\sum}_{i}^{64}{\left(\frac{{N}_{i}^{{{{\rm{img}}}}}}{{\sum}_{j}^{64}{N}_{j}^{{{{\rm{img}}}}}}-\frac{1}{64}\right)}^{2}}$$

3 $${X}_{{{{\rm{m}}}}}=\frac{64\cdot \max \left({N}^{{{{\rm{img}}}}}\right)}{S}$$

where ${N}^{{{{\rm{img}}}}}$ is the number of adhesion cells counted from each image, $S$ is the surface area of the 15.3 mm diameter space, ${X}_{{{{\rm{seed}}}}}$ is the seeding density (see also Table 1), and $\max \left(\cdot \right)$ is the function that obtains the maximum value.

**Table 1:** Description of symbols, Greek letters, and acronyms

Symbols		
$A$	–	Quality specification
${CDS}$	–	Correctly identified feasible condition
$C\overline{{DS}}$	–	Correctly identified infeasible condition
$f$	–	Kinetic model function
$h$	%	Probability based on prediction
${h}^{\exp }$	%	Probability based on experiment
$I$	–	Indicator function
${IDS}$	–	Incorrectly identified feasible condition
$I\overline{{DS}}$	–	Incorrectly identified infeasible condition
${K}_{{{{\rm{s}}}}}$	–	Spatial limitation constant
${L}^{n}$	–	Parameter combination
$M$	–	Number of model iterations
$n$, ${n}_{{{{\rm{sample}}}}}$	–	Number of samples
${n}_{{{{\rm{measure}}}}}$	–	Number of measurements
$n{CDS}$	–	Number of correctly identified feasible conditions
${nC}\overline{{DS}}$	–	Number of correctly identified infeasible conditions
${nIDS}$	–	Number of incorrectly identified feasible conditions
${nI}\overline{{DS}}$	–	Number of incorrectly identified infeasible conditions
$N$	–	Number of adhesion cells
${N}^{\exp }$	–	Number of experimentally measured adhesion cells
${N}^{{{{\rm{img}}}}}$	–	Number of adhesion cells counted in an image
$N{|}_{{pi}}$	–	Limits of the predicted number of adhesion cells
$N{|}_{{pi}={{{\rm{UP}}}}}$, $N{|}_{{pi}={{{\rm{LO}}}}}$	–	Upper and lower limits of the predicted number of adhesion cells
${pi}$	–	Prediction interval
$P$	–	Confluency level
${P}^{\exp }$	–	Experimentally measured confluency level
$P{|}_{{pi}}$	–	Limits of the predicted confluency level
$P{|}_{{pi}={{{\rm{UP}}}}}$, $P{|}_{{pi}={{{\rm{LO}}}}}$	–	Upper and lower limits of the predicted confluency level
${R}_{1}$	–	Ratio of conditions included in the DS that are feasible to the total number of conditions in the DS
${R}_{2}$	–	Ratio of the number of feasible conditions correctly included in the DS to the total number of feasible conditions
${R}_{3}$	–	Ratio of the number of infeasible conditions correctly included in the DS to the total number of infeasible conditions
$s$	–	Sample standard deviation
$S$	cm^2^	Surface area
$t$	h	Cultivation time
${t}_{{{{\rm{h}}}}}$	day	Harvesting time
${t}_{\left(p,r\right)}$	–	$p$ quantile of Student’s t distribution with $r$ degrees of freedom
$X$	cells cm^−2^	Adhesion cell density
${X}_{0}$	cells cm^−2^	Cell density on Day 1
${X}_{{{{\rm{m}}}}}$	cells cm^−2^	Maximum cell density
${X}_{{{{\rm{seed}}}}}$	cells cm^−2^	Seeding cell density
$X{|}_{{pi}}$	–	Limits of the predicted adhesion cell density
$X{|}_{{pi}={{{\rm{UP}}}}}$, $X{|}_{{pi}={{{\rm{LO}}}}}$	cells cm^−2^	Upper and lower limits of the predicted adhesion cell density
$x$	–	Design variable
${{{\boldsymbol{y}}}}{|}_{{pi}}$	–	Limits of the model predictions
${{{\boldsymbol{y}}}}{|}_{{pi}={{{\rm{UP}}}}}$, ${{{\boldsymbol{y}}}}{|}_{{pi}={{{\rm{LO}}}}}$	–	Upper and lower limits of the model predictions
**Greek letters**		
$\alpha$	–	Adhesion ratio
$\varepsilon$	–	Seeding heterogeneity
${{{\boldsymbol{\theta }}}}$	–	Parameter matrix
$\mu$	h^−1^	Specific growth rate
${\mu }_{{{{\rm{m}}}}}$	h^−1^	Maximum specific growth rate
${\mu }_{{{{\rm{m}}}}}^{{{{\rm{UP}}}}}$, ${\mu }_{{{{\rm{m}}}}}^{{{{\rm{LO}}}}}$	h^−1^	Upper and lower bounds of the prediction interval of the maximum specific growth rate
$\pi$	%	Minimum acceptable risk
**Acronyms**		
CMA		Critical material attribute
CPP		Critical process parameter
CQA		Critical quality attribute
DS		Design space
Exp 1		Initial experiment
Exp 2A		Second experiment by operator A
Exp 2B		Second experiment by operator B
Exp 2C		Second experiment by operator C
LO		Lower bound of a prediction interval
MSC		Mesenchymal stem cell
NRMSE		Normalized root mean square error
NRMSE(fit)		NRMSE for the model fit
NRMSE(initial)		NRMSE for the initial model prediction
NRMSE(validation)		NRMSE for the model validation
ODE		Ordinary differential equation
PDMS		Polydimethylsiloxane
PSE		Process systems engineering
QbD		Quality by design
RSS		Residual sum of squares
UP		Upper bound of a prediction interval

### Model formulation

The previous kinetic model of MSC cultivation processes with a set of ordinary differential equations (ODEs)^26^ was applied in this work. Specifically, the following three equations were formulated to describe time-dependent changes in adhesion cell density on the basis of the specific growth rate while considering spatial limitation and contact inhibition as follows^26^:

4 $$\frac{{dX}}{{dt}}=\mu X$$

5 $${X}_{0}=\alpha {X}_{{{{\rm{seed}}}}}$$

6 $$\mu ={\mu }_{{{{\rm{m}}}}}\left(1-\varepsilon {K}_{{{{\rm{s}}}}}\right)\left(1-\frac{X}{{X}_{{{{\rm{m}}}}}}\right)$$

where $X$ is the adhesion cell density at a given cultivation time, $t$; ${X}_{0}$ is the cell density on Day 1; $\alpha$ is the adhesion ratio; $\varepsilon$ is the seeding heterogeneity; $\mu$ is the specific growth rate; ${\mu }_{{{{\rm{m}}}}}$ is the maximum specific growth rate; and ${K}_{{{{\rm{s}}}}}$ is the spatial limitation constant (see also Table 1). The *scipy.integrate.solve_ivp* solver was used with its default parameters for solving the ODEs in Python 3.9.13 via an Intel Xeon Gold 6142 CPU @ 2.60 GHz with 128 GB of RAM.

### Estimation of the maximum specific growth rate

The experimental data were used to estimate the value of ${\mu }_{{{{\rm{m}}}}}$ in the kinetic models. The value of ${K}_{{{{\rm{s}}}}}$ was 24.7 [–] from the literature^26^, whereas the other parameter values in the equations (i.e., $\alpha$, $\varepsilon$, and ${X}_{{{{\rm{m}}}}}$) were set as the means of the experimental data (Table S2). To use the least squares method, the residual sum of squares (RSS)^41^ was calculated for each sample as follows:

7 $${RS}{S}_{i} = {\sum}_{j=1}^{{n}_{{{{\rm{measure}}}}}}{\left({N}_{i,j}^{\exp }-{N}_{i,j}\right)}^{2}$$

where ${RR}{S}_{i}$ is the calculated RSS for a given sample; ${N}_{i,j}^{\exp }$ is the measured number of adhesion cells; ${N}_{i,j}$ is the predicted number of adhesion cells; and ${n}_{{{{\rm{measure}}}}}$ is the number of measurements (see also Table 1). The RSS was subsequently minimized to obtain the value of ${\mu }_{{{{\rm{m}}}}}$ for each sample, which yielded their mean value as the estimate of ${\mu }_{{{{\rm{m}}}}}$. This minimization was performed with the *scipy.optimize.minimize* function by setting its solver as *Nelder‒Mead*, its maximum number of iterations as 5000, and its other parameters as defaults in Python 3.9.13.

To evaluate the errors between the experimental data and the model prediction, the NRMSE^41^ was calculated for each seeding density as follows:

8 $${NRMS}{E}_{k}=\frac{\sqrt{\frac{1}{{n}_{{{{\rm{sample}}}}}\cdot {n}_{{{{\rm{measure}}}}}}{\sum}_{i=1}^{{n}_{{{{\rm{sample}}}}}}{\sum }_{j=1}^{{n}_{{{{\rm{measure}}}}}}{\left({N}_{i,j}^{\exp }-{N}_{i,j}\right)}^{2}}}{\max \left({N}_{k}^{\exp }\right)-\min \left({N}_{k}^{\exp }\right)}\times 100 \%$$

where ${NRMS}{E}_{k}$ is the calculated NRMSE for a given seeding density; ${n}_{{{{\rm{sample}}}}}$ is the number of samples (see also Table 1); and $\max \left({N}_{k}^{\exp }\right)$ and $\min \left({N}_{k}^{\exp }\right)$ are the maximum and minimum values of the measured number of adhesion cells for a given seeding density, respectively.

### Model validation

The model was validated by confirming an NRMSE between the model prediction and the remaining experimental data for each seeding density of less than a prior threshold of 10%^18^. With respect to the model inputs, the parameter values of $\alpha$, $\varepsilon$, and ${X}_{{{{\rm{m}}}}}$ were the same as those used for the ${\mu }_{{{{\rm{m}}}}}$ parameter estimation. In addition, the estimated ${\mu }_{{{{\rm{m}}}}}$ was used as a model input.

### Prediction interval calculation

Two-sided 95% prediction intervals^30^ of the estimated ${\mu }_{{{{\rm{m}}}}}$ were calculated as follows:

9 $${\mu }_{{{{\rm{m}}}}}^{{{{\rm{UP}}}}}={\mu }_{{{{\rm{m}}}}}+{t}_{\left(0.975,n-1\right)}s\sqrt{1+\frac{1}{n}}$$

10 $${\mu }_{{{{\rm{m}}}}}^{{{{\rm{LO}}}}}={\mu }_{{{{\rm{m}}}}}-{t}_{\left(0.975,n-1\right)}s\sqrt{1+\frac{1}{n}}$$

where ${\mu }_{{{{\rm{m}}}}}^{{{{\rm{UP}}}}}$ and ${\mu }_{{{{\rm{m}}}}}^{{{{\rm{LO}}}}}$ are the upper and lower bounds of the prediction interval of ${\mu }_{{{{\rm{m}}}}}$, respectively; ${t}_{ (p,r )}$ is the $p$ quantile of the Student’s t distribution with $r$ degrees of freedom; $s$ is the sample standard deviation of ${\mu }_{{{{\rm{m}}}}}$; and $n$ is the number of samples (see also Table 1). Here, ${t}_{\left(p,r\right)}$ was calculated with the default *t* test function implemented in the *scipy* library in Python 3.9.13.

### Dynamic and stochastic simulation

Given a set seeding density and harvesting time, time-dependent changes in cell growth were simulated until day 9 at the end of cultivation (i.e., dynamic simulation). Variations in the growth kinetics were predicted by determining the upper and lower limits; specifically, ${\mu }_{{{{\rm{m}}}}}^{{{{\rm{UP}}}}}$ and ${\mu }_{{{{\rm{m}}}}}^{{{{\rm{LO}}}}}$ were substituted into ${\mu }_{{{{\rm{m}}}}}$ in Eq. (6), respectively. Additionally, to account for the effects of other sources of variation, such as operation variability, on time-dependent changes in cell density and confluency, the input parameters (i.e., $\alpha$, $\varepsilon$, and ${X}_{{{{\rm{m}}}}}$) were randomly selected from the individual experimental measurements (Table S2) and substituted into the kinetic model. This dynamic simulation was repeated with random sampling of the parameters (i.e., dynamic and stochastic simulation) until the outputs converged; specifically, relative standard deviations of the mean of the final number of adhesion cells were evaluated at the end of the iteration. Here, the sampling was performed with the default *numpy.random.choice* function implemented in Python 3.9.13.

### Design space determination

The probabilistic DS was determined on the basis of the dynamic and stochastic simulation results to ensure the following quality specifications for the number of adhesion cells and confluency level, which were the same as those in prior work^26^.

11 $$A=\left\{\left(N,P\right)\left|5.0\times {10}^{4}\le N\wedge P \, < \, 0.8\right.\right\}$$

where $A$ is the set of quality specifications; $N$ is the number of adhesion cells; and $P$ is the confluency level (see also Table 1). Here, a previously described algorithm^18,21^ was used to calculate the probability to define a DS as follows:

12 $$h\left({{{\boldsymbol{CPP}}}}\right)=\frac{1}{M}{\sum}_{i=1}^{M}I\left({{{{\boldsymbol{y}}}}}_{i}\in A\right)\times 100 \%$$

where $h$ is the calculated probability; ${{{\boldsymbol{CPP}}}}$ is a set of CPPs; $I\left(\cdot \right)$ is an indicator function, which takes a value of 1 if all conditions are satisfied and 0 if at least one condition is not satisfied; $M$ is the number of model iterations for a given design variable; and ${{{\boldsymbol{y}}}}$ is the model prediction (see also Table 1).

In this work, the abovementioned algorithm was extended with a probability calculation using the limits of growth predictions as follows:

13 $${DS}=\left\{\left(x,t\right)\in {L}^{n}\left|h\left(x,t\right)\ge \pi \right.\right\}$$

s.t.

14 $$h\left(x,t\right)=\frac{1}{M}{\sum}_{i=1}^{M}I\left({{{{\boldsymbol{y}}}}}_{i}{|}_{{pi}={{{\rm{UP}}}}}\in A\wedge {{{{\boldsymbol{y}}}}}_{i}{|}_{{pi}={{{\rm{LO}}}}}\in A\right)\times 100 \%$$

where $\left(x,t\right)$ is a set of seeding densities and harvesting times among the parameter combinations, ${L}^{n}$; $\pi$ is a user-specified minimum acceptable risk; and ${{{{\boldsymbol{y}}}}|}_{{pi}={{{\rm{UP}}}}}$ and ${{{{\boldsymbol{y}}}}|}_{{pi}={{{\rm{LO}}}}}$ are the upper and lower limits of the model predictions, respectively (see also Table 1). Here, both limits contained the predicted outputs (i.e., number of adhesion cells, confluency level) on the basis of the prediction interval of ${\mu }_{{{{\rm{m}}}}}$; specifically, the outputs were calculated with the adhesion cell density, which was a function of seeding density and harvesting time as follows:

15 $${{{\boldsymbol{y}}}}{|}_{{pi}}=\left(N{|}_{{pi}},P{|}_{{pi}}\right)$$

16 $$N{|}_{{pi}}={SX}{|}_{{pi}}$$

17 $$P{|}_{{pi}}=\frac{X{|}_{{pi}}}{{X}_{{{{\rm{m}}}}}}$$

18 $$X{|}_{{pi}}=\left\{\begin{array}{c}f\left(x,t,{\mu }_{{{{\rm{m}}}}}^{{{{\rm{UP}}}}},{{{\boldsymbol{\theta }}}}\right),{pi}={{{\rm{UP}}}}\\ f\left(x,t,{\mu }_{{{{\rm{m}}}}}^{{{{\rm{LO}}}}},{{{\boldsymbol{\theta }}}}\right),{pi}={{{\rm{LO}}}}\end{array}\right.$$

where $f$ is the kinetic model; ${{X|}}_{{pi}}$ represents the limits of the predicted adhesion cell density; ${{N|}}_{{pi}}$ represents the limits of the predicted number of cells; ${{P|}}_{{pi}}$ represents the limits of the predicted confluency level; and ${{{\boldsymbol{\theta }}}}$ represents an $M\times$ 3 matrix containing the values of three input parameters (i.e., $\alpha$, $\varepsilon$, and ${X}_{{{{\rm{m}}}}}$) (see also Table 1).

### Design space validation

The conditions proposed on the basis of the DS results were experimentally validated to confirm whether the actual cultivation results met the specifications. The measurements in the validation experiment were used to calculate this probability, ${h}^{\exp }$.

19 $${h}^{\exp }=\frac{1}{n}{\sum}_{i=1}^{n}I\left({N}_{i}^{\exp }\in A\wedge {P}_{i}^{\exp }\in A\right)\times 100 \%$$

where $n$ is the number of samples in the validation experiment for a given condition; ${N}^{\exp }$ is the measured number of adhesion cells; and ${P}^{\exp }$ is the confluency estimated from the measurements (see also Table 1).

$h$ and ${h}^{\exp }$ were analyzed to categorize all the investigated cultivation conditions (i.e., sets of seeding density and harvesting time) into one of four categories as follows:

- ${CDS}$: correctly identified feasible condition, in which both $h$ and ${h}^{\exp }$ are equal to or greater than $\pi$.
- $C\overline{{DS}}$: correctly identified infeasible condition, in which both $h$ and ${h}^{\exp }$ are less than $\pi$.
- ${IDS}$: incorrectly identified feasible condition, in which $h$ is equal to or greater than $\pi$ but ${h}^{\exp }$ is less than $\pi$.
- $I\overline{{DS}}$: incorrectly identified infeasible condition, in which $h$ is less than $\pi$ but ${h}^{\exp }$ is equal to or greater than $\pi$.

In addition to these definitions, the following three metrics were defined to evaluate the DS.

- ${R}_{1}$: the ratio of the number of conditions included in the DS that were feasible in the validation experiment to the total number of conditions in the DS.
- ${R}_{2}$: the ratio of the number of feasible conditions in the validation experiment that were correctly included in the DS to the total number of feasible conditions.
- ${R}_{3}$: the ratio of the number of infeasible conditions in the validation experiment that were correctly included in the DS to the total number of infeasible conditions.

21 $${R}_{1}=\frac{{nCDS}}{{nCDS}+{nIDS}}$$

22 $${R}_{2}=\frac{{nCDS}}{{nCDS}+{nI}\overline{{DS}}}$$

23 $${R}_{3}=\frac{{nC}\overline{{DS}}}{{nC}\overline{{DS}}+{nIDS}}$$

where ${nCDS}$, ${nC}\overline{{DS}}$, ${nIDS}$, and ${nI}\overline{{DS}}$ are the numbers of conditions classified as ${CDS}$, $C\overline{{DS}}$, ${IDS}$, or $I\overline{{DS}}$, respectively (see also Table 1). If the values of ${R}_{1}$, ${R}_{2}$, and ${R}_{3}$ are all close to 1, the model-based DS can be considered to provide robust cultivation conditions. In particular, ${R}_{1}$ represents how conservatively the DS proposes feasible conditions, which suggests that a high ${R}_{1}$ value would enable cell manufacturers to safely select feasible conditions from the DS.

### Statistics and reproducibility

All available data from the cultivation experiments were used, and no statistical method was employed to determine the sample size. No data were excluded from the analysis. The cultivation experiment with each condition was replicated six times independently. Three sets of samples were allocated with every permutation. The authors performed every permutation and, therefore, were not blinded to the sample allocation.

### Reporting summary

Further information on research design is available in the Nature Portfolio Reporting Summary linked to this article.

## Supplementary information

Supplementary information

Supplementary Data 1

Supplementary Data 2

Supplementary Data 3

Supplementary Data 4

Supplementary Data 5

Description of Additional Supplementary Materials

Reporting summary

## Data Availability

All data supporting the findings of this study are available within the article and Supplementary Information. The raw data from the experiments and the numerical source data are also available from Supplementary Data 1 and Supplementary Data 2–5, respectively.

## Code availability

The code used in this study can be obtained from https://github.com/UTokyoPSE/Hirono_etal_DSValidation.git.
