# Supplementary material for: Determination and validation of design space for mesenchymal stem cell cultivation processes using prediction intervals
Source: Commun Biol. 2025 May 8;8:657. doi: 10.1038/s42003-025-08063-2 (PMC12062477; doi:10.1038/s42003-025-08063-2)
Supplement: Supplementary file 1 — Supplementary information [file 42003_2025_8063_MOESM1_ESM.pdf]

Supplementary Material

Determination and validation of design space for mesenchymal stem cell cultivation processes using prediction intervals

Keita Hirono[1], Yusuke Hayashi[1], Isuru A. Udugama[1], Mohamed Rami Gaddem[1], Kenjiro Tanaka[2], Yuto Takemoto[2], Ryuji Kato[2,3], Masahiro Kino-oka[4], and Hirokazu Sugiyama[1,*]

[1]*Department of Chemical System Engineering, The University of Tokyo, 7-3-1, Hongo, Bunkyo-ku, Tokyo 113-8656, Japan*

[2]*Department of Basic Medicinal Sciences, Graduate School of Pharmaceutical Sciences, Nagoya University, Tokai National Higher Education and Research System, Furocho, Chikusa-ku, Nagoya, Aichi 464-8601, Japan*

[3]*Institute of Nano-Life-Systems, Institutes of Innovation for Future Society, Nagoya University, Tokai National Higher Education and Research System, Furocho, Chikusa-ku, Nagoya, Aichi 464-8601, Japan*

[4]*Department of Biotechnology, Osaka University, 1-2, Yamadaoka, Suita-shi, Osaka 565-0871, Japan*

[*]Corresponding author

sugiyama@chemsys.t.u-tokyo.ac.jp

TEL & FAX: +81-3-5841-7227

Supplementary Results

Conventional design space determination

The prior kinetic model[1] was applied to the conventional method[2] to determine a DS. First, ordinary differential equations[1] were used to simulate growth kinetics while considering spatial limitations and contact inhibition in mesenchymal stem cell (MSC) cultivation. The maximum specific growth rate, μ_{m}, was subsequently estimated from the number of adhesion cells measured in Exp 1, where the seeding density, X_{seed}, was 2000 cells cm^{-2}. The least squares optimization method yielded an μ_{m} of 4.02 × 10^{-2} h^{-1} and a normalized root mean square error (NRMSE) of 5.21%, indicating good agreement between the model and the measurements in Exp 1 (Fig. S1a). Second, the estimated μ_{m} was used to simulate the number of adhesion cells over time (i.e., Days 1–9) as a function of X_{seed} and the harvesting time, t_{h}. Additionally, stochastic simulation[1] was conducted by randomly sampling values of other model parameters (e.g., the adhesion ratio, seeding heterogeneity, and maximum cell density) from the measurements in the first experiment (Exp 1) to consider likely growth variations. The resulting dynamic variations in the growth prediction were quantified for a X_{seed} of 3000 cells cm^{-2} (Fig. S1b). The simulation conditions were subsequently extended to X_{seed} of interest (i.e., 1500–4500 cells cm^{-2}), such that conditions with X_{seed} values of 1500, 3000, and 4500 cells cm^{-2} could be experimentally validated with data from the second experiment (Exp 2). Third, a DS was visualized as a set of X_{seed} and t_{h} where the simulation results met the quality specifications with a probability of 90% or greater (Fig. S1c). Through this DS, quantitative and concrete conditions were specified and used for DS validation.

Insufficient validation of the conventional design space

The second set of experiments (Exps 2A–2C), in which MSCs from the same lot as those used in Exp 1 were used to validate the DS, was performed to investigate the seeding conditions

both inside and outside the DS. X_{seed} was set as 1500, 3000, and 4500 cells cm^{-2} (a total of 3 densities), and t_{h} ranged from Days 1 to 9 in 6 h increments (a total of 33 measurement points). To assess the practical applicability of the DS estimated from the model constructed via Exp 1 data in real-world cell manufacturing scenarios, we designed an experimental dataset that intentionally incorporated bias and noise typically encountered in cell cultivation processes. The variability inherent in cell cultivation, particularly with MSCs, presents a significant challenge, as the outcomes can differ slightly with each culture attempt. A model that fails to account for this variability would be unsuitable for practical application. Consequently, we conducted Exp 2, which was designed to closely resemble Exp 1 but with key differences, such as the involvement of three distinct operators. Each operator's experiment was treated as an independent study, with $n = 6$ (i.e., 6 wells) replicates per experiment. X_{seed} and t_{h} were defined from Exp 1. For each X_{seed} and t_{h}, a total of 18 samples were investigated by three operators (Operators A, B, and C; each analyzing 6 samples) to calculate the ratio of samples out of the 18 samples that satisfied the quality specifications, which yielded 99 probabilities (3 densities × 33 measurements).

DS validation metrics were established on the basis of model accuracy metrics in previous work[3]. Although the prior metrics evaluated a surrogate model against the original function[3], we compared the calculated DS with the experimental results. Specifically, the calculated and measured probabilities were compared among the 99 conditions to categorize the investigated conditions into four categories, where CDS is a correctly identified feasible condition, $\overline{CDS}$ is a correctly identified infeasible condition, IDS is an incorrectly identified feasible condition, and $\overline{IDS}$ is an incorrectly identified infeasible condition (Fig. 1). The validation of the DS in Fig. S1c was conducted in terms of these four categories. The validation against the data of Exps 2A–2C failed because all conditions (i.e., X_{seed} and t_{h}) inside the DS were identified as IDS, with many $\overline{IDS}$ conditions found outside the DS (Fig. S1d). To

investigate the sources of the insufficient validation results, the model predictions and the corresponding measurements in Exps 2A–2C were compared for all X_{seed} values, which yielded NRMSEs larger than 20% (Fig. S2). These results indicated that the fitting parameter of the model (μ_{m}) needed to be re-estimated to incorporate sources of errors, including growth variability, before determining the DS.

Impact of the sample size of pre-experiments on the model and design space

The impact of the sample size employed in the pre-experiment on the predictive capacities of the model and DSs was evaluated utilizing the Exp 2 data. Specifically, NRMSEs were calculated for the model, while the validation categories and metrics were quantified for the DSs. In this evaluation, six different levels of sample numbers, n, were investigated as a sum of the investigation with the three seeding densities ($X_{\mathrm{seed}} = 1500$, 3000, 4500 cells cm^{-2}) by a single operator, yielding a total of three to 18 samples with an interval of three samples (i.e., $n = 3, 6, \ldots, 18$) for each operator.

For a given set of X_{seed} and operator, NRMSEs for the number of adhesion cells were evaluated by comparing the pre-experimental observations and the model prediction (Fig. S5). Specifically, the mean and standard deviation of the NRMSEs were calculated from all possible combinations (Fig. S5). While the NRMSEs for the fitting remained relatively constant, those for the validation improved as n increased (Fig. S5). Comparing the upper bound of the error bars for the validation results with a given accuracy criterion of 10%, a minimum acceptable sample size was determined as twelve ($n = 12$). Subsequently, among the specified acceptable sizes ($n \geq 12$), DS validation results were obtained for a given set of π (50–90%) and operator as illustrated in Fig. S6 ($n = 12, 15$) and Fig. 6 ($n = 18$). To achieve a given precision criterion of $R_1 \geq 0.9$, a sample size of 18 was required. This result indicated that an increase in the sample size ensured the specification of the input parameter distributions and prediction

intervals, thus enhancing the reliability of the resulting DSs.

Figure legends

Fig. S1| Insufficient validation results for the conventional design space.

Fig. S2| Comparison of model predictions before parameter re-estimation with measurements from validation experiments.

Fig. S3| Re-estimation of the maximum specific growth rate and limits of the growth prediction results with different pre- and validation experiments.

Fig. S4| Design space determination results with different pre-experiments.

Fig. S5| Impacts of the sample size of pre-experiments on predictive capacities of the model with different pre-experiments.

Fig. S6| Impacts of the sample size of pre-experiments on design space validation results with different pre-experiments.

Table S1| Estimated values of the maximum specific growth rate.

Table S2| Calculated parameter values.

Table S3| Design space validation metrics without/with prediction interval calculation.

Table S4| Design space validation metrics for different pre- and validation experiments.

Table S5| Impacts of the sample size of pre-experiments on design space validation metrics for different pre- and validation experiments.

Fig. S1| Insufficient validation results for the conventional design space. a. Model fit to the mean of the experimental number of adhesion cells from Exp 1 (dashed line). The error bars show the means and standard deviations of the six samples. The gray scatters represent the individual samples. The normalized root mean square error (NRMSE) is annotated on the plot. **b**. Dynamic and stochastic simulation. The model prediction for the seeding density of 3000 cells cm^{-2} was iterated 1000 times (light black lines). **c**. Design space determination. The contour map shows the predicted probability as a function of seeding density and harvesting time with a design space (DS) boundary where the probability is 90% (black lines). **d**. Design space validation. The black lines show the DS with the resulting outcomes out of $\boldsymbol{IDS}$, incorrectly identified feasible condition (red dots); $\boldsymbol{C\overline{DS}}$, correctly identified infeasible

condition (blue); and $\overline{\boldsymbol{IDS}}$, incorrectly identified infeasible condition (yellow).

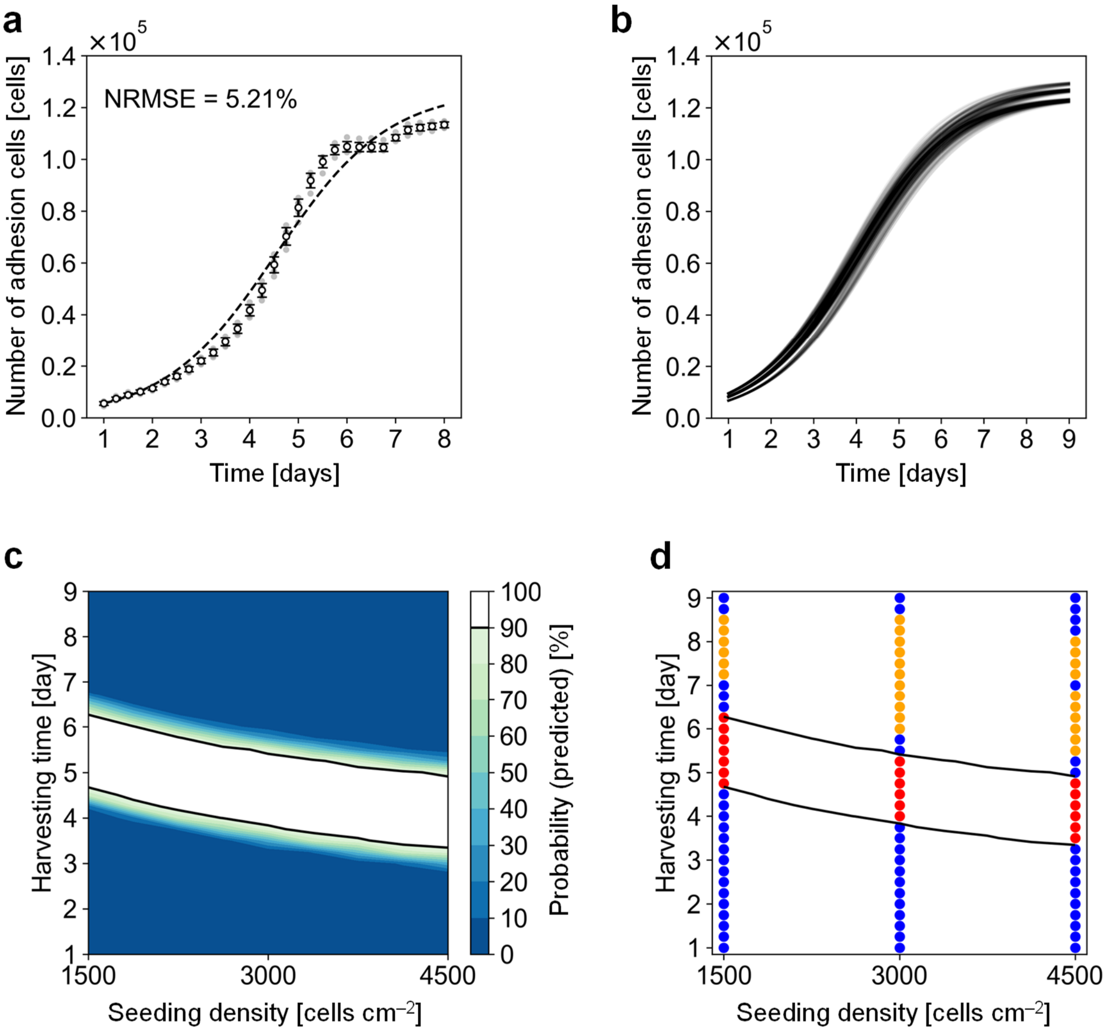

Fig. S2| Comparison of model predictions before parameter re-estimation with measurements from validation experiments. The initial model prediction was compared with the experimental number of adhesion cells from Exps 2A–2C for a given seeding density (black lines). The error bars show the means and standard deviations of the 18 samples in Exps 2A–2C. The gray scatters represent the individual samples. The normalized root mean square error between the mean of the number of adhesion cells from Exps 2A–2C and the initial model prediction (NRMSE(initial)) is annotated on the plot.

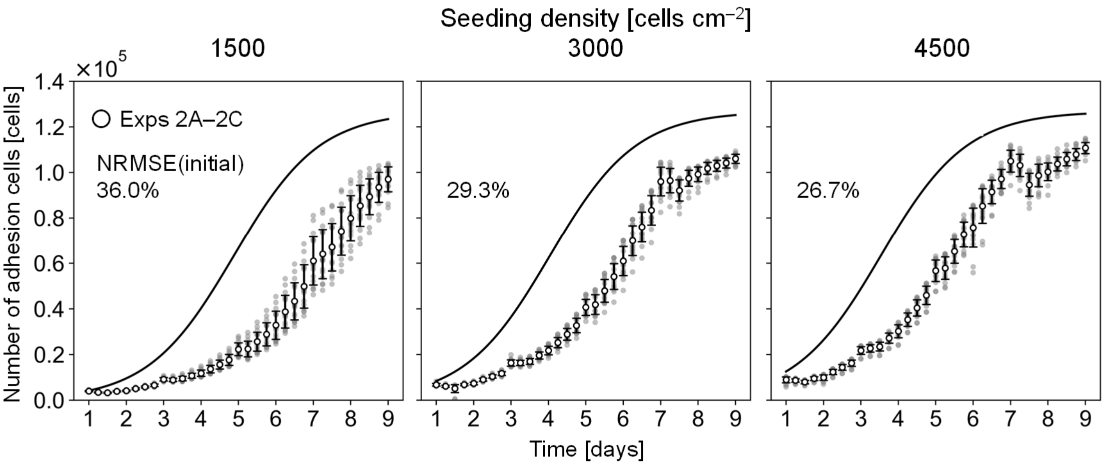

Fig. S3| Re-estimation of the maximum specific growth rate and limits of growth prediction results with different pre- and validation experiments. a. Exp 2A and Exps 2B & 2C were used for the pre-experiment and validation experiments, respectively. **b**. Exp 2B and Exps 2C & 2A were used for the pre-experiment and validation experiments, respectively. **c**. Exp 2C and Exps 2A & 2B were used for the pre-experiment and validation experiments, respectively. Here, the parameter was re-estimated (dashed red line) via the pre-experimental results (first column), the model was validated (orange line) via the validation experimental results (second), and the upper (green) and lower (blue) prediction limits were compared with the validation experimental results (third) for a seeding density of 3000 cells cm^{-2}. The normalized root mean square error (NRMSE) for the model fit (NRMSE(fit)) and validation (NRMSE(validation)) are annotated on the plot. The error bars for the fitting (first column), validation (second), and prediction limits (third) represent the means and standard deviations of the six, twelve, and twelve samples, respectively. The gray scatters represent the individual samples.

155

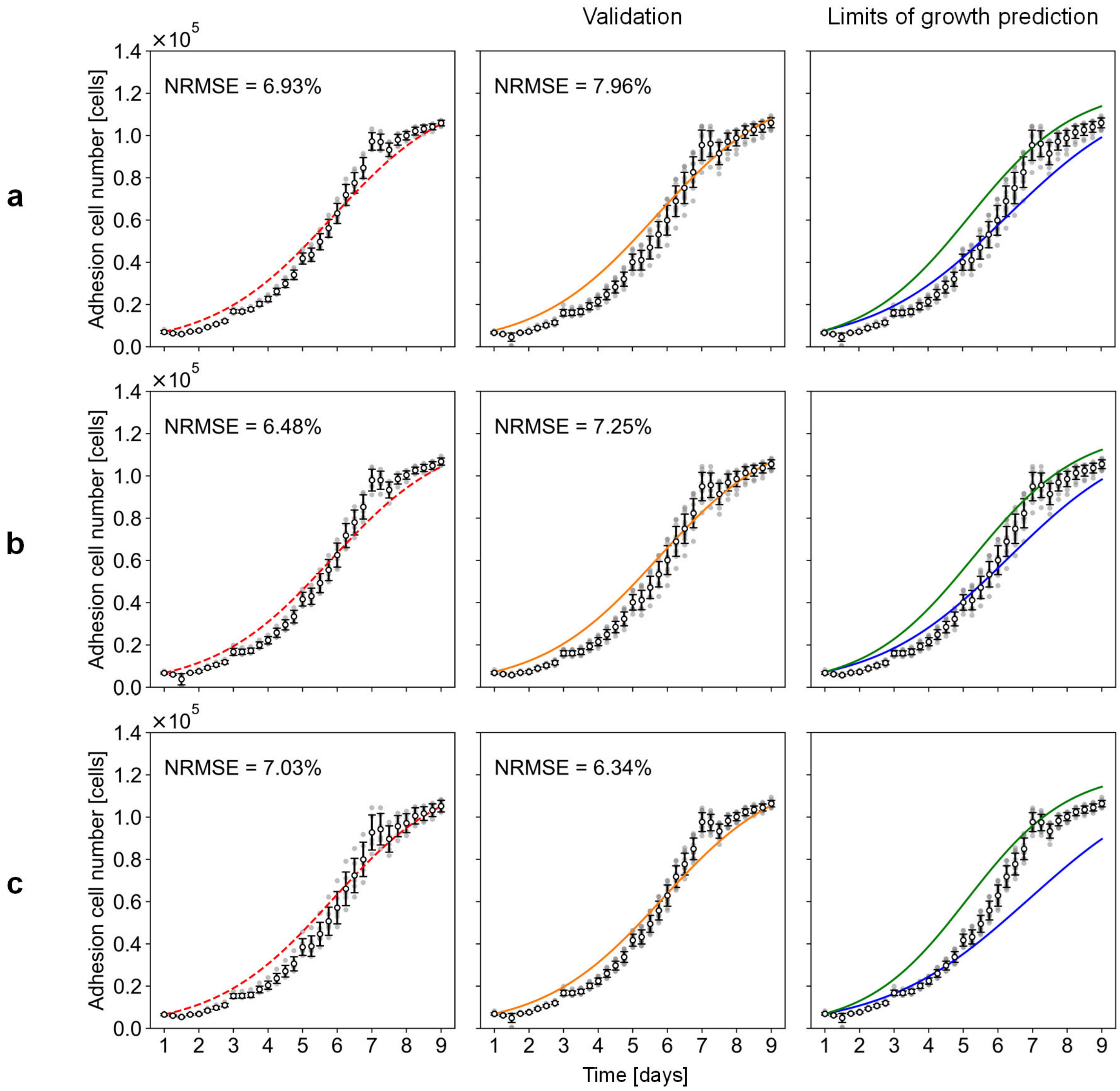

Validation
Limits of growth prediction
a
b
c
Adhesion cell number [cells]
×10^5
NRMSE = 6.93%
NRMSE = 7.96%
NRMSE = 6.48%
NRMSE = 7.25%
NRMSE = 7.03%
NRMSE = 6.34%
Time [days]

Fig. S4| Design space determination results with different pre-experiments. The design spaces were determined via pre-experimental results for a given minimum acceptable risk, $\boldsymbol{\pi}$. The contour map shows the predicted probability as a function of seeding density and harvesting time with the design space boundary, where the probability is equal to $\boldsymbol{\pi}$ (black lines). **a**. Exp 2A was used for the pre-experiment. **b**. Exp 2B was used for the pre-experiment. **c**. Exp 2C was used for the pre-experiment.

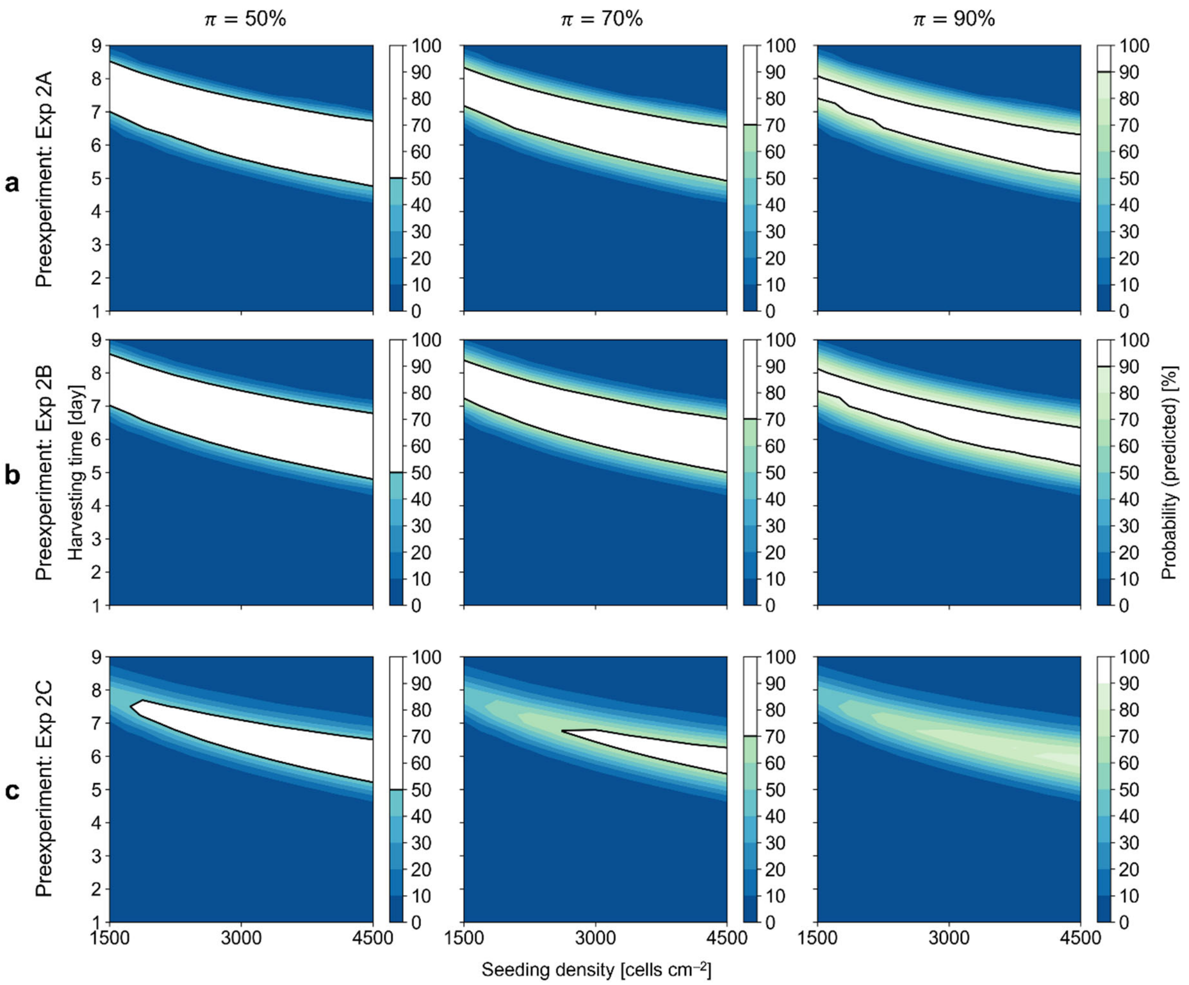

Fig. S5| Impacts of the sample size of pre-experiments on predictive capacities of the model with different pre-experiments. The normalized root mean square error (NRMSE) between the mean of the number of adhesion cells from pre-experiments and the model prediction was evaluated. The error bars show the standard deviations of NRMSEs for a given number of samples. The gray scatters represent the individual NRMSEs. The dashed line (green) shows an accuracy criterion of 10%. **a**. Model fitting results (NRMSE(fit)). **b**. Model validation results (NRMSE(validation)).

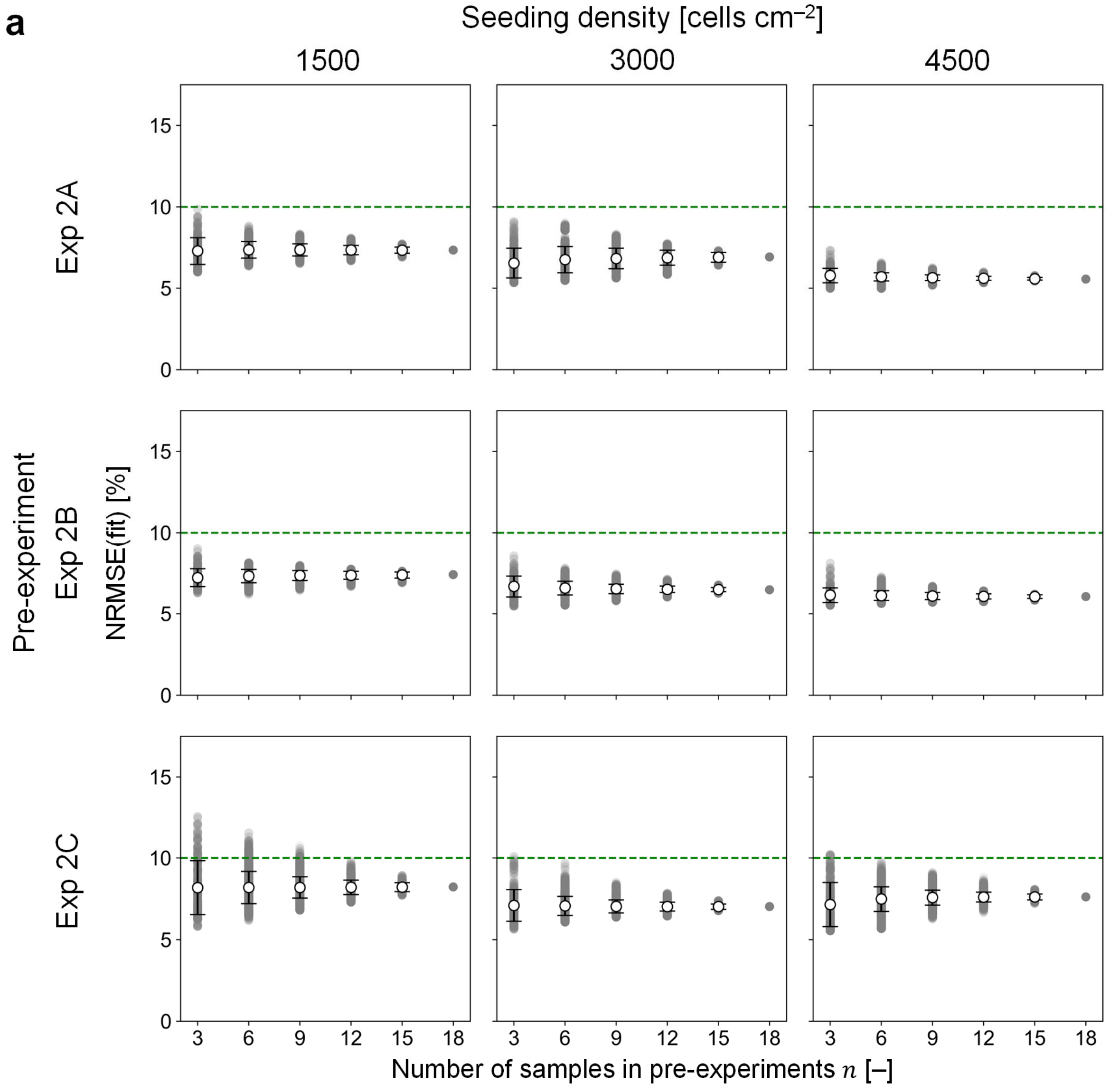

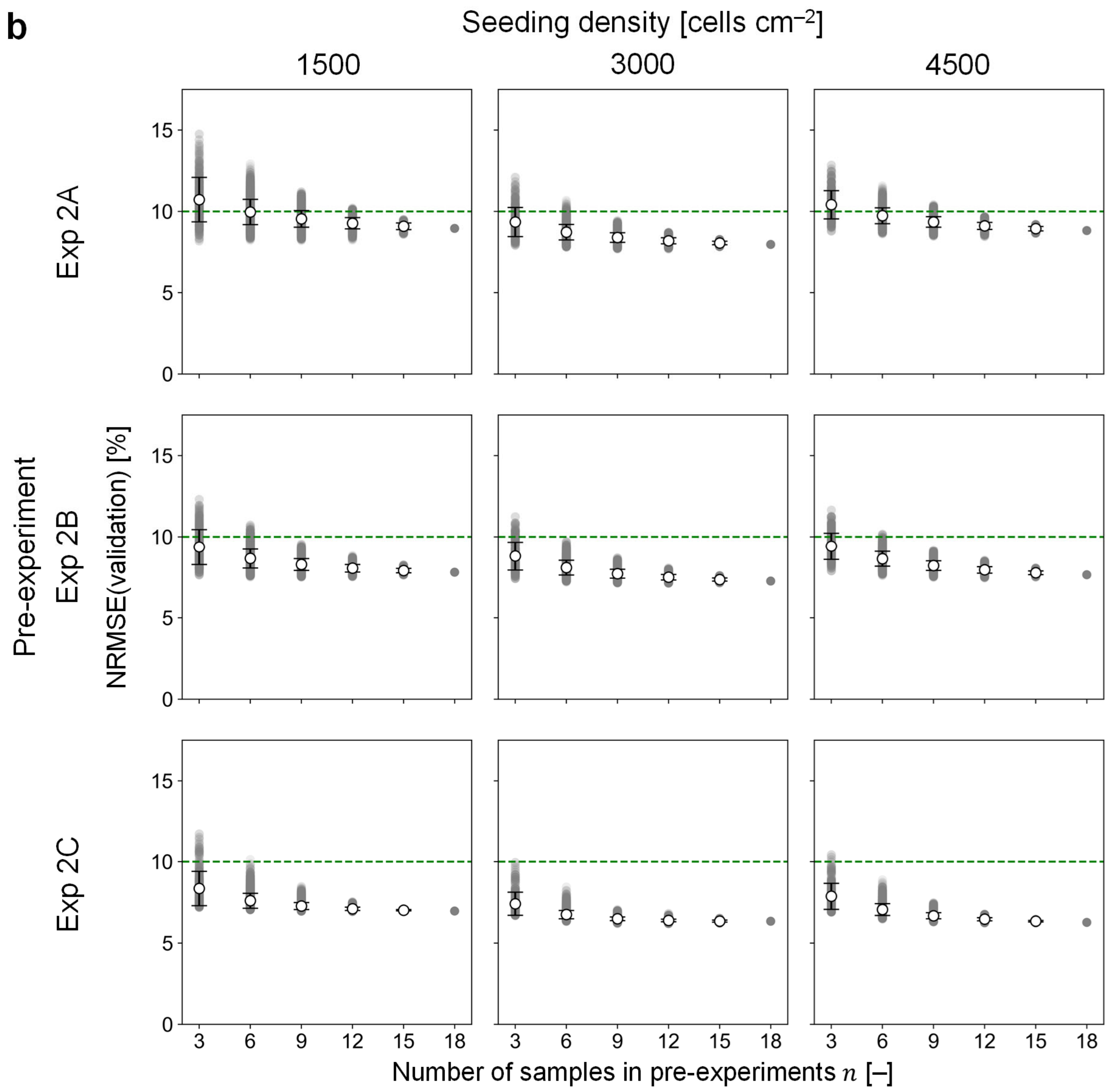
b
Seeding density [cells cm⁻²]
1500
3000
4500
Exp 2A
Exp 2B
Exp 2C
Pre-experiment
NRMSE(validation) [%]
Number of samples in pre-experiments n [–]

172

Fig. S6| Impacts of the sample size of pre-experiments on design space validation results with different pre-experiments. The design spaces were determined using a given number of samples in the pre-experiments and then validated with the validation experimental results for a given minimum acceptable risk, $\boldsymbol{\pi}$. The black lines represent the DS with the resulting categories out of $\boldsymbol{CDS}$, correctly identified feasible condition (green dots); $\boldsymbol{IDS}$, incorrectly identified feasible condition (red); $\overline{\boldsymbol{CDS}}$, correctly identified infeasible condition (blue); and $\overline{\boldsymbol{IDS}}$, incorrectly identified infeasible condition (yellow). **a**. Twelve samples were used for the pre-experiment. **b**. A total of 15 samples were used for the pre-experiment.

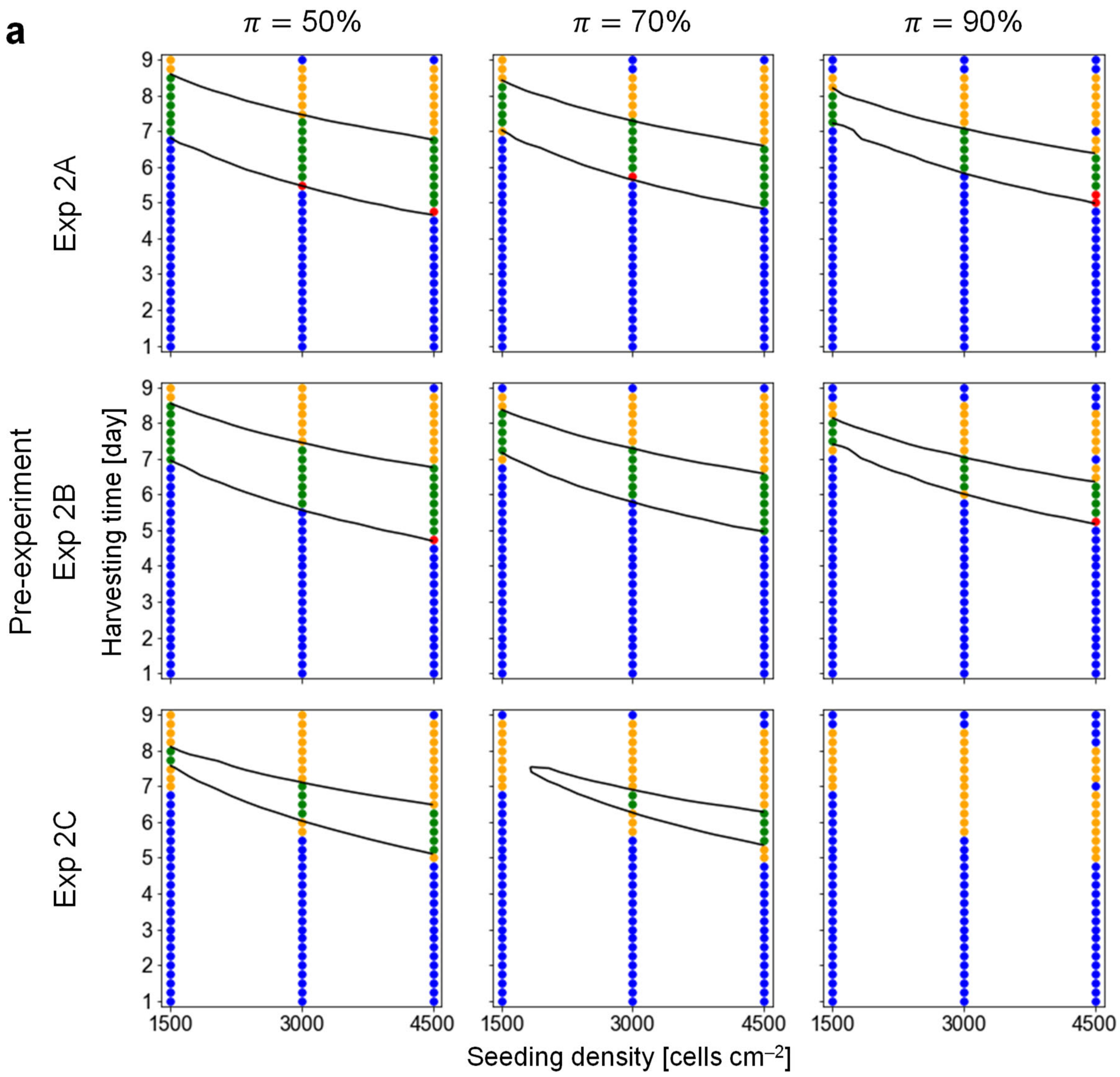

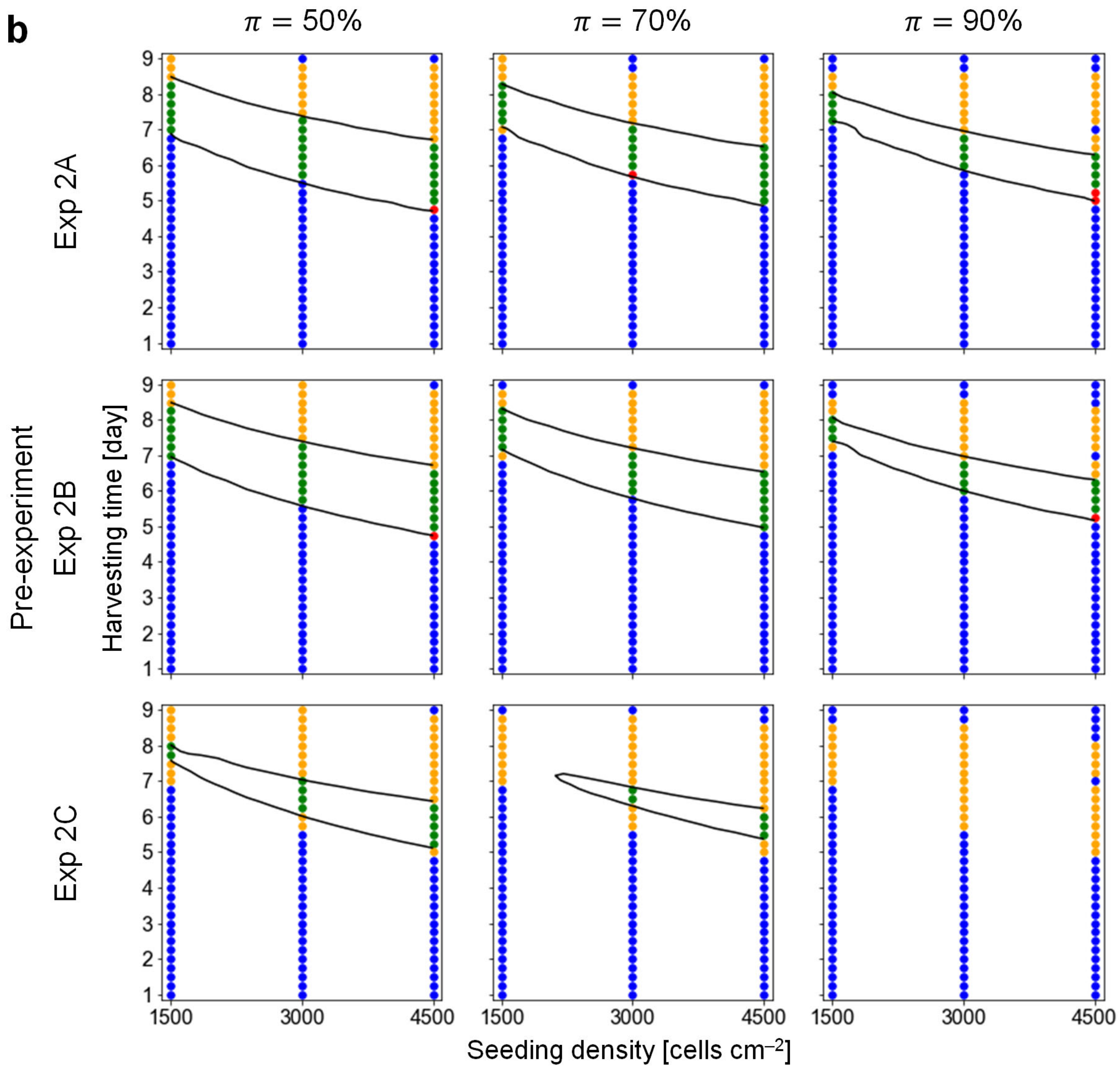
b
π = 50%
π = 70%
π = 90%
Exp 2A
Pre-experiment
Exp 2B
Exp 2C
Harvesting time [day]
Seeding density [cells cm⁻²]
1500
3000
4500

182

Table S1| Estimated values of the maximum specific growth rate. SD: sample standard deviation.

Seeding density [cells cm^{-2}]	Well ID	Maximum specific growth rate [10^{-2} h^{-1}]		
		Exp 1		
2000	1	3.87		
	2	3.96		
	3	4.26		
	4	4.12		
	5	3.99		
	6	3.94		
Mean of the six samples		4.02		
		Exp 2A	Exp 2B	Exp 2C
1500	1	2.64	2.62	2.47
	2	2.98	2.88	2.81
	3	2.59	2.55	2.41
	4	2.72	2.62	2.52
	5	2.81	2.99	2.86
	6	2.53	2.51	2.55
3000	1	2.77	2.81	2.63
	2	2.91	2.91	2.85
	3	2.74	2.73	2.50
	4	2.79	2.84	2.70
	5	3.07	2.84	2.83
	6	2.54	2.71	2.49
4500	1	2.66	2.54	2.54
	2	2.80	2.70	2.96
	3	2.63	2.66	3.04
	4	2.79	2.80	2.63
	5	2.87	2.83	3.10
	6	2.75	2.76	3.04
Mean of the 18 samples		2.76	2.74	2.72

SD of the 18 samples	0.146	0.136	0.222

Table S2| Calculated parameter values.

Seeding density [cells cm^{-2}]	Well ID	Adhesion ratio [–]			Seeding heterogeneity [10^{-3}]			Maximum cell density [10^{4} cells cm^{-2}]		
					Exp 1					
2000	1	1.36			4.31			5.49		
	2	1.34			4.59			5.48		
	3	0.956			2.82			5.31		
	4	1.15			5.12			5.60		
	5	1.17			3.22			5.33		
	6	1.19			4.01			5.31		
Mean of the six samples		1.19			4.01			5.42		
					Exp 2					
		2A	2B	2C	2A	2B	2C	2A	2B	2C
1500	1	1.32	1.13	1.06	6.65	4.50	4.76	5.02	4.82	5.18
	2	1.18	1.07	1.15	5.40	5.37	5.08	5.06	5.02	4.92
	3	1.19	1.11	1.11	5.30	5.82	4.71	4.90	5.16	4.78
	4	1.10	1.12	1.07	6.56	5.66	4.54	5.23	5.03	5.01
	5	1.29	1.12	1.12	4.96	6.27	5.71	5.24	5.02	5.04
	6	1.27	1.06	1.02	5.61	7.15	6.39	5.01	4.88	4.79
3000	1	1.00	0.897	0.906	5.36	4.81	4.90	5.32	5.39	5.53
	2	1.01	1.09	1.02	5.09	5.31	4.45	5.27	5.15	5.33
	3	0.953	0.954	1.02	5.20	4.73	3.72	5.26	4.99	5.07
	4	0.940	0.946	0.874	4.95	4.46	3.17	5.30	5.24	5.08
	5	0.943	0.929	0.864	6.19	3.09	4.16	5.16	5.09	5.36
	6	1.18	0.956	0.918	4.78	3.94	2.77	5.18	5.01	4.80
4500	1	0.969	0.867	0.891	4.38	2.71	3.18	5.59	5.45	5.40
	2	0.936	0.895	0.662	4.34	3.03	2.38	5.37	5.22	5.11
	3	0.945	0.861	0.549	4.90	4.09	5.41	5.35	5.39	5.22
	4	0.968	0.819	0.855	5.31	4.47	4.27	5.33	5.28	5.35

5	0.929	0.878	0.784	5.04	4.88	6.60	5.40	5.70	5.57
6	0.960	0.924	0.629	5.50	4.51	7.39	5.24	5.24	5.47
Mean of the 18 samples	1.06	0.979	0.916	5.31	4.71	4.64	5.23	5.17	5.17
Mean of all the 24 samples	1.09	1.03	0.986	4.98	4.54	4.49	5.28	5.31	5.23

Table S3| Design space validation metrics without/with prediction interval calculation.

	$nCDS$ [–]	$nIDS$ [–]	$nC\overline{DS}$ [–]	$nI\overline{DS}$ [–]	R_1 [–]	R_2 [–]	R_3 [–]
Without interval calculation	19	8	62	10	0.704	0.655	0.886
With interval calculation	11	1	69	18	0.917	0.379	0.986

Table S4| Design space validation metrics for different pre- and validation experiments.

Pre-experiment/validation experiments	π [%]	$nCDS$ [–]	$nIDS$ [–]	$nC\overline{DS}$ [–]	$nI\overline{DS}$ [–]	R_1 [–]	R_2 [–]	R_3 [–]
2A/2B & 2C	50	20	0	61	18	1.00	0.526	1.00
	70	17	0	63	19	1.00	0.472	1.00
	90	11	1	69	18	0.917	0.379	0.986
2B/2C & 2A	50	21	0	60	18	1.00	0.538	1.00
	70	17	0	63	19	1.00	0.472	1.00
	90	11	1	70	17	0.917	0.393	0.986
2C/2A & 2B	50	10	0	60	29	1.00	0.256	1.00
	70	6	0	63	30	1.00	0.167	1.00
	90	0	0	68	31	–	0.000	1.00

Table S5| Impacts of the sample size of pre-experiments on design space validation metrics for different pre- and validation experiments.

n [–]	Pre-experiment/ validation experiments	π [%]	$nCDS$ [–]	$nIDS$ [–]	$nC\overline{DS}$ [–]	$nI\overline{DS}$ [–]	R_1 [–]	R_2 [–]	R_3 [–]
12	2A/2B & 2C	50	22	2	59	16	0.92	0.58	0.97
		70	18	1	62	18	0.95	0.50	0.98
		90	13	2	68	16	0.87	0.45	0.97
	2B/2C & 2A	50	22	1	59	17	0.96	0.56	0.98
		70	18	0	63	18	1.00	0.50	1.00
		90	11	1	70	17	0.92	0.39	0.99
	2C/2A & 2B	50	11	0	60	28	1.00	0.28	1.00
		70	6	0	63	30	1.00	0.17	1.00
		90	0	0	68	31	–	0.00	1.00
15	2A/2B & 2C	50	20	1	60	18	0.95	0.53	0.98
		70	17	1	62	19	0.94	0.47	0.98
		90	12	2	68	17	0.86	0.41	0.97
	2B/2C & 2A	50	20	1	59	19	0.95	0.51	0.98
		70	17	0	63	19	1.00	0.47	1.00
		90	11	1	70	17	0.92	0.39	0.99
	2C/2A & 2B	50	11	0	60	28	1.00	0.28	1.00
		70	5	0	63	31	1.00	0.14	1.00
		90	0	0	68	31	–	0.00	1.00

Nomenclature

Symbols		
CDS	–	Correctly identified feasible condition
$C\overline{DS}$	–	Correctly identified infeasible condition
IDS	–	Incorrectly identified feasible condition
$I\overline{DS}$	–	Incorrectly identified infeasible condition
n	–	Number of samples
$nCDS$	–	Number of correctly identified feasible condition
$nC\overline{DS}$	–	Number of correctly identified infeasible condition
$nIDS$	–	Number of incorrectly identified feasible condition
$nI\overline{DS}$	–	Number of incorrectly identified infeasible condition
R_1	–	Ratio of conditions included in the DS that are feasible to the total number of conditions in the DS
R_2	–	Ratio of the number of feasible conditions correctly included in the DS to the total number of feasible conditions
R_3	–	Ratio of the number of infeasible conditions correctly included in the DS to the total number of infeasible conditions
t_{h}	day	Harvesting time
X_{seed}	cells cm^{-2}	Seeding cell density
Greek letters		
μ_{m}	h^{-1}	Maximum specific growth rate

π	%	Minimum acceptable risk
Acronyms		
DS		Design space
Exp 1		Initial experiment
Exp 2A		Second experiment by operator A
Exp 2B		Second experiment by operator B
Exp 2C		Second experiment by operator C
MSC		Mesenchymal stem cell
NRMSE		Normalized root mean square error
NRMSE(fit)		NRMSE for the model fit
NRMSE(initial)		NRMSE for the initial model prediction
NRMSE(validation)		NRMSE for the model validation

References

1. Hirono, K. *et al.* Image‐based hybrid model incorporating initial spatial distribution for mesenchymal stem cell cultivation process design. *AIChE J.* **70**, e18452 (2024).
2. Hirono, K., A. Udugama, I., Hayashi, Y., Kino-oka, M. & Sugiyama, H. A dynamic and probabilistic design space determination method for mesenchymal stem cell cultivation processes. *Ind. Eng. Chem. Res.* **61**, 7009–7019 (2022).
3. Wang, Z. & Ierapetritou, M. A novel feasibility analysis method for black‐box processes using a radial basis function adaptive sampling approach. *AIChE J.* **63**, 532–550 (2017).
