# Supplementary material for: Determination and validation of design space for mesenchymal stem cell cultivation processes using prediction intervals
Source: Commun Biol. 2025 May 8;8:657. doi: 10.1038/s42003-025-08063-2 (PMC12062477; doi:10.1038/s42003-025-08063-2)
Supplement: Supplementary file 7 — Description of Additional Supplementary Materials [file 42003_2025_8063_MOESM7_ESM.pdf]

Description of Additional Supplementary Files

File name: Supplementary Data 1

Description: raw data from the experiments

File name: Supplementary Data 2-5

Description: the numerical source data
